# Transcriptional and Functional Programming of Decidual Innate Lymphoid Cells

**DOI:** 10.3389/fimmu.2019.03065

**Published:** 2020-01-24

**Authors:** Jessica Vazquez, Deborah A. Chasman, Gladys E. Lopez, Chanel T. Tyler, Irene M. Ong, Aleksandar K. Stanic

**Affiliations:** ^1^Division of Reproductive Sciences, Department of Obstetrics and Gynecology, University of Wisconsin-Madison, Madison, WI, United States; ^2^Department of Biostatistics and Medical Informatics, University of Wisconsin-Madison, Madison, WI, United States; ^3^Division of Maternal-Fetal Medicine, Department of Obstetrics and Gynecology, University of Wisconsin-Madison, Madison, WI, United States; ^4^Carbone Comprehensive Cancer Center, University of Wisconsin-Madison, Madison, WI, United States; ^5^Division of Reproductive Endocrinology and Infertility, Department of Obstetrics and Gynecology, University of Wisconsin-Madison, Madison, WI, United States

**Keywords:** ILCs, decidua, t-SNE, pregnancy, transcription factors, cytokines

## Abstract

A successful pregnancy requires many physiological adaptations from the mother, including the establishment of tolerance toward the semiallogeneic fetus. Innate lymphoid cells (ILCs) have arisen as important players in immune regulation and tissue homeostasis at mucosal and barrier surfaces. Dimensionality reduction and transcriptomic analysis revealed the presence of two novel CD56^Bright^ decidual ILCs that express low T-bet and divergent Eomes levels. Transcriptional correlation with recently identified first trimester decidual dNKs suggests that these novel decidual ILCs might be present throughout pregnancy. Functional testing with permutation analysis revealed production of multiple factors by individual cells, with a preference for IFNγ and VEGF. Overall, our data suggests continuity of a unique decidual innate lymphocytes across pregnancy with a polyfunctional functional profile conducive for pregnancy

## Introduction

Innate lymphoid cells (ILCs), lymphocytes that lack antigen-specific receptors, have been implicated in immune regulation and tissue homeostasis at mucosal and barrier surfaces (1). Recent studies have classified three groups within the ILC family, based on cytokine profiles and transcription factor expression (2). Type 1 ILCs, characterized by the expression of T-bet (1) and secretion of IFNγ (3); type 2 ILCs, characterized by the expression of GATA-3 and secretion of IL-5, IL-9, IL-13 (4, 5); and type 3 ILCs, including a lymphoid tissue inducer (LTi)-like subset, characterized by the expression of RORγt and secretion of both IL-17 and L-22 (2, 6). Furthermore, natural killer cells (NKs), now part of the ILC family, are dependent on both T-bet and Eomes and secrete IFNγ (7), although tissue-resident NK (trNK) cells have tissue-specific T-bet/Eomes requirements (8).

Pregnancy requires many physiological adaptations from the mother. In particular, the maternal immune system needs to establish tolerance toward the semiallogeneic fetus, while maintaining the ability to fight infections that might pose a threat to the mother and the fetus. Furthermore, decidual NK (dNKs) cells have been implicated in early pregnancy processes involving the guidance of trophoblast invasion as well as angiogenesis (9). Interestingly, mice deficient in the transcription factor *nfil3*, necessary for conventional NK (cNK) development, show only partial decidual defects and retain ability to produce viable pups (10). Deeper examination of *nfil3*^0/0^ mice demonstrated that they retain trNK in the liver (8), cells of a similar phenotype in the uterus (8, 11, 12), and a small proportion in the salivary glands (13). This data suggests that dNKs are more closely related, if not, uterine trNK cells, a conjecture supported by the examination of dNK cells by classical DBA-stain histology in the uterus (14) and their expansion from local progenitors (15). Subset complexity might also help explain why dNKs have not been definitively linked to pregnancy pathologies (16, 17).

Recent advances in data analysis now allow mapping of tissue-specific immunomes, aiding in the identification of rare cellular subsets. We have recently used dimensionality reduction, tSNE, coupled with clustering, DensVM, to map both T cells and dendritic cells of term human decidua (18) and track immune changes across murine gestation (19). By leveraging the power of these methods onto RNA sequencing and functional testing, we identified two novel human subsets of CD56^Bright^CD16^−^ decidual ILCs (dILCs). Further analysis revealed that these two subsets, despite differing in Eomes expression, have similar functional profiles, but distinct gene expression signatures. Re-analysis of our data in the context of recent single cell sequencing-based map of first trimester decidua (20) suggests continuity of dILC subsets across pregnancy—from first trimester through term decidua. Taken together, we demonstrate a high level of the heterogeneity of CD56^Bright^CD16^−^ ILCs, revealing additional targets for understanding pregnancy pathology.

## Materials and Methods

### Human Samples

De-identified term human (>37 wks GA) placental samples were collected from normal elective cesarean sections under the UW Obstetrical Tissue Bank IRB protocol (#2014-1223) and UnityPoint Health—Meriter IRB protocol (#2017-004; Supplementary Table 1). Briefly, decidua basalis was separated from placenta and decidua parietalis was scraped from the fetal membrane and washed with cold PBS, as previously described (21). Tissue was minced with scissors in RPMI containing 1 mg/ml of Collagenase type V (Worthington Biochem. Corp.), 2 μg/ml DNAse I (Worthington Biochem. Corp.). Tissues were then dissociated using the gentleMACS^™^ Dissociator system (Miltenyi Biotec Inc. San Diego, CA). Briefly, tissues were placed in a gentleMACS^™^ C Tube and an in-house program was developed for tissue dissociation (starting with 10 loops: clockwise spin of 100 rpm for 1 min, counter-clockwise spine of 100 rpm for 1 min; followed by a clockwise spin of 1,000 rpm for 5 s; then 5 loops: clockwise spin of 100 rpm for 1 min, counter-clockwise spine of 100 rpm for 1 min; ending with a clockwise spin of 1,000 rpm for 5 s; all steps run at 37°C) and run on the gentleMACS^™^ Dissociator for a total of 30 min. Homogenates were then filtered through a 70 μm filter, red blood cells were lysed with ACK lysis buffer (Life Technologies) for 5 min and mononuclear cells (MCs) were recovered and frozen in DMSO with 10% FBS until processing. All experiments included technical controls, from batched, anonymous, non-pregnant, reproductive age female PBMCs (All Cells^®^, Alameda, CA) and kept frozen until processing.

### Flow Cytometry and Standardization

Isolated MCs were first labeled with LIVE/DEAD^®^ fixable blue stain (Invitrogen) or Zombie NIR^™^ (BioLegend) in PBS for 15 min on ice. MCs were then labeled with flourochrome-conjugated monoclonal antibodies, listed in Supplementary Table 2. Briefly, antibodies were diluted in BD Horizon Brilliant^™^ Stain Buffer (BD Biosciences, San Jose, CA) and used to label MCs for 30 min on ice. Transcription factor assessment was performed using BD Pharmigen^™^ Transcription Factor Buffer Set, with permeabilization of cells occurring overnight, followed by antibody labeling for 50 min on ice. Cytokine production assessment was performed using BD Cytofix/Cytoperm^™^ according to manufacturer's instructions. Samples were acquired using the LSR Fortessa in a 5 laser (355, 405, 488, 562, 633 nm) 20-detector configuration (BD Biosciences).

SPHERO^™^ Rainbow Calibration Particles (Sperotech, Lake Forest, IL) were used to standardize PMT voltage settings. Briefly, PMT voltages were optimized during first experimental run. Median flourscence intensity (MFI) values were then calculated for the Rainbow beads and were used in subsequent experimental runs as target values (±10%) to set PMT voltages.

### Innate Lymphoid Cell Isolation

Prior to FACS sorting, ILCs were enriched using Dynabeads^™^ Untouched^™^ Human NK Cells Kit (Invitrogen), allowing for the depletion of T, B, DCs, and macrophages. Enriched ILCs were labeled with flourochrome-conjugated monoclonal antibodies, listed in Supplementary Table 2, and C10 (CD3^−^CD14^−^CD19^−^CD34^−^CD56^Bright^CD94^+^CD16^−^CD127^−^ CD49a^−^), C2 (CD3^−^CD14^−^CD19^−^CD34^−^CD56^Bright^CD94^+^ CD16^−^CD127^−^CD49a^+^), and cNK (CD3^−^CD14^−^CD19^−^ CD34^−^ CD56^Dim^CD94^+^CD16^+^), were sorted using the BD FACS Aria II (BD Biosciences) into fetal bovine serum for culture and activation or lysing buffer (NucleoSpin^®^ RNA XS, Takara Bio USA Inc, Mountain View, CA) for RNA isolation.

### Innate Lymphoid Cell Activation

Sorted ILCs were incubated for 24 h in complete culture media (RPMI with 10% FBS, 1% Pen/Strep, 2 mM Glutamine, 25 mM HEPES) supplemented with premixed PMA/Ionomycin (Cell Activation Cocktail without Brefeldin A, BioLegend, cat#423301, Supplementary Table 3) according to manufacturer's instructions. Supernatants were then collected and processed using the LEGENDPlex^™^ (BioLegend) to assess the range of cytokine and growth factor production.

In a separate set of experiments, MCs were incubated in complete culture media, supplemented with a combination of IL-12 (50 ng/mL), IL-15 (50 ng/mL), IL-1β (50 ng/mL), IL-18 (50 ng/mL), or with IL-23 (10 ng/mL) (all acquired from BioLegend; Supplementary Table 3) for 18 h, or with premixed PMA/Ionomycin (Leukocyte Activation Cocktail with BD GolgiPlug^™^, cat#550583,BD Bioscience, Supplementary Table 3) for 6 h, according to manufacturer's instructions. For retention of cytokines, MCs were treated with BD GolgiPlug^™^ (Brefeldin A) and BD GolgiStop^™^ (Monensin) (BD Bioscience) for the last 6 h of the activation period. Cytokine production was then assessed as described above.

### RNAseq and qPCR

#### RNA Isolation, QC, Library Preparation, and Sequencing

MCs were sorted from the decidua (pooled basalis and parietalis) directly into lysing buffer, as described above, and lysates were processed using the NucleoSpin^®^ RNA XS (Takara Bio USA Inc) according to manufacturer's instructions. A small aliquot of RNA from each sample was set aside for RNA quality control and quantification. RNA quality was assessed using the Agilent^©^ RNA 6000 Pico Kit and analyzed on the Agilent^©^ 2100 Bioanalyzer. RNA quantity was measured using the NanoDrop^™^ Spectrophotometer (Thermo Scientific^™^). Libraries were prepared using the SMART-Seq^®^ v4 Ultra^®^ Low Input RNA Kit for Sequencing (Takara Bio USA Inc) and sequenced on the Illumina^©^ HiSeq 2500 system.

#### qPCR

cDNA was generated using the High-Capacity cDNA Reverse Transcription Kit (Thermo Scientific^™^) according to manufacturer's instructions. PCR reaction cocktail was prepared for each sample using the TaqMan^®^ Gene Expression PCR Master Mix (Thermo Scientific^™^) and sample cDNA, according to manufacturer's instructions. PCR reaction cocktail was then mixed with TaqMan^®^ Gene Expression Assay (Thermo Scientific^™^) for target genes Eomes (Hs00172872_m1), VEGFA (Hs00900055_m1), and 18S (Hs99999901_s1) and run on the Applied Biosystem^™^ Thermocycler (Thermo Scientific^™^), following manufacturer's instructions. Samples were run in triplicates, with biological replicates indicated in figure legends. Data was then analyzed using the StepOne^™^ Software (v.3).

### Data Analysis

#### Dimensionality Reduction and Cluster Assignment

Manual analysis identifying well-characterized populations was performed using FlowJo v.10 software (FlowJo LLC, Ashland, OR). Dimensionality reduction was performed using the t-SNE algorithm, followed by DensVM clustering, both part of the open-source R package, Cytofkit (22). Data was pre-processed as previously described (18). Briefly, data files were pre-gated to exclude dead cells and irrelevant populations, downsampled using FlowJo internal downsampling tool (5000 cells per sample), and concatenated using FlowJo. Concatenated files were then uploaded to R/Cytofkit via GUI interface and parameters of interest were selected. Newly derived t-SNE and DensVM coordinates were added to original data matrices, exported, and analyzed in FlowJo. Cluster frequencies and MFI values were then calculated within FlowJo. Heatmaps for MFI (z-score normalized) and cluster frequencies were constructed using JMP Pro^®^ v. 11.0.0 (SAS, Cary, NC). Cell ontology was implemented within FlowJo for cluster classification (23).

#### RNA-Seq Data Processing

We used Skewer to trim Illumina adapters and low-quality 3′ ends with score < 20, and kept resulting reads of at least 30 bp. We performed alignment and quantification using the STAR pipeline within RSEM (v1.3.1) software. Within RSEM, reads were first aligned to the annotated human reference genome GRCh38 (release 94) with STAR (v2.6.1a) using parameter settings outFilterMultimapNmax=20, outFilterMismatchNoverLmax=0.04, alignIntronMin=20. Gene expression levels were quantified as expected counts (ECs). To identify and remove un-expressed genes, we rescaled ECs to counts per million (CPM) and accepted genes with mean CPM > 0 across samples.

Diagnostic plots [inter-sample correlation and principle component analysis (PCA)] were generated with utilities from DESeq2 (v1.22.2) on variance-stabilized counts. PCA was performed using the top 500 most varying genes. One sample (Decidua 062-Cluster 10) was determined to be an outlier because the second major principal component, explaining 20% of the variance, clearly separated this sample from all others. The sample was also the least correlated to all other samples, including the other C10 samples. After removing the outlier and filtering unexpressed genes, 15,672 genes were available for downstream analysis. The PCA plot shown in Figure 2A was computed without the outlier sample.

#### Differential Expression Analyses

To obtain consensus of differentially expressed genes, we interrogated the dataset by three methods: edgeR, DEseq2, and EBSeq. RNA-seq data were provided to each method as specified in the respective documentation.

edgeR: ECs were rounded to integer values, transformed to CPM, and normalized by Trimmed Mean of M-values (TMM). We defined the design matrix as ~0+Celltype+Decidua, which accounts for batch effect by decidua. Including 0+ ensures that the design matrix will include columns for all three cell types, which is required to perform the desired tests. Differential expression analyses were performed by fitting and testing a quasi-likelihood negative binomial generalized log-linear model (glmQLFTest). The expression in each cell type was compared to the mean of the other two using contrasts of the form 1A – 0.5B – 0.5C = 0. For example, for A = cNK, B = C2, C = C10, the contrast denotes a comparison of cell type cNK to the mean of C2 and C10. DE genes were defined at FDR < 0.05.

DESeq2: We supplied ECs rounded to integer values. The DESeq2 model was fit using the design matrix ~Celltype+Decidua and betaPrior=TRUE. This design matrix is functionally identical to the model supplied to edgeR, but in DESeq2, zero-intercept models are incompatible with the beta prior parameter. We performed the same contrasts as for edgeR, and defined DE genes at FDR < 0.05.

EBSeq: We applied EBSeq to the ECs using scripts included with RSEM. Given three cell types, EBSeq assesses the likelihood of each gene following one of five patterns: same expression in all cell types (Pattern 1, not DE), different between one cell type and the other two (Patterns 2-4, DE), or different in all cell types (Pattern 5, DE). Genes are scored by the posterior probability of belonging to any differential expression pattern (PPDE), where FDR = 1-PPDE. We called DE genes with FDR <0.05 and assigned each to its respective maximum a posteriori pattern. Compared to edgeR and DESeq2 results, Patterns 2–4 are analogous to the contrasts performed by edgeR and DESeq2. Unlike those methods, the DE gene sets from EBSeq are disjoint, and the method cannot account for batch effects.

#### Comparison to dNK Subsets

Vento-Tormo et al. (20) defined three decidual NK subtypes based on shared nearest-neighbor cluster analysis of single cell RNA-seq from first trimester decidua (dNK1-3). We obtained differentially expressed genes for each dNK population from Supplementary Table 7 with *q* < 0.05, and further defined “marker genes” for each cell type as described in the caption for Figure 4A (20). Marker genes for a cell population were positively expressed (log fold change >0), expressed in at least 10% of cells in the population, and expressed in fewer than 60% of other cells. Applying these rules yielded 135 genes specific to dNK1, 45 genes for dNK2, and 208 genes for dNK3.

We compared the ILC populations to the dNK populations using two analyses. First, for all genes that were differentially expressed in each dNK population (regardless of ILC DE status), we assessed the similarity of their log fold change rankings to the log fold changes in the ILC populations using Spearman correlation. Significant correlations shown in Figure 4A were called at FDR <0.05, having adjusted for multiple testing using the Benjamini-Hochberg procedure. Second, we tested for enrichment of each dNK population's marker genes among the up-regulated DE genes for each ILC population using hypergeometric tests. The threshold for a significant overlap shown in Figure 4B was defined at FDR <0.05.

#### Gene Set Enrichment Analysis

We applied GOSeq (24) to test the DE gene sets for each cell type for enrichment of externally produced gene sets (or “terms”). Enrichment was defined at FDR <0.05, determined by applying the Benjamini–Hochberg procedure to all individual tests for the set of terms. We selected gene sets for visualization in Figure 3 by taking the union of the top 5 enriched terms for each DE gene set by adjusted *p*-value, then filtering for gene sets that were identified by at least two algorithms or for two different cell types.

We obtained the three Gene Ontologies (25, 26) using the R package GO.db (27). We accessed KEGG pathway annotations from the R package org.Hs.eg.db (28). From the Molecular Signatures Database (29, 30), we obtained gene sets for binding motifs of transcription factors (31) and immunologic gene sets derived from microarray studies (32).

#### Functional Attributes

Boolean gates were drawn in FlowJo to determine the proportion of cells that were positive for any given combination of cytokines or growth factor. SPICE (33) was then used to visualize the polyfunctional properties of novel decidual ILCs.

The integrated Mean Fluorescence Intensity (iMFI) was calculated as previously described (34, 35) to quantify total functional response. Briefly, iMFI was calculated by multiplying the frequency of cytokine-positive cells by the MFI.

#### Statistical Comparisons

Statistical significance was determined with Student's *t*-test or ANOVA analysis, followed by Tukey's *post-hoc* test to correct for multiple comparisons, using Prism^®^ v. 7 (GraphPad Software, Inc, La Jolla, CA) or JMP Pro^®^. Pie chart comparisons were conducted using the permutation (Monte Carlo) testing within SPICE. All statistical information regarding comparisons are found in Supplementary Tables 5–13.

## Results

### Innate Lymphoid Cells in Human Term Decidua

**Figure 1:**
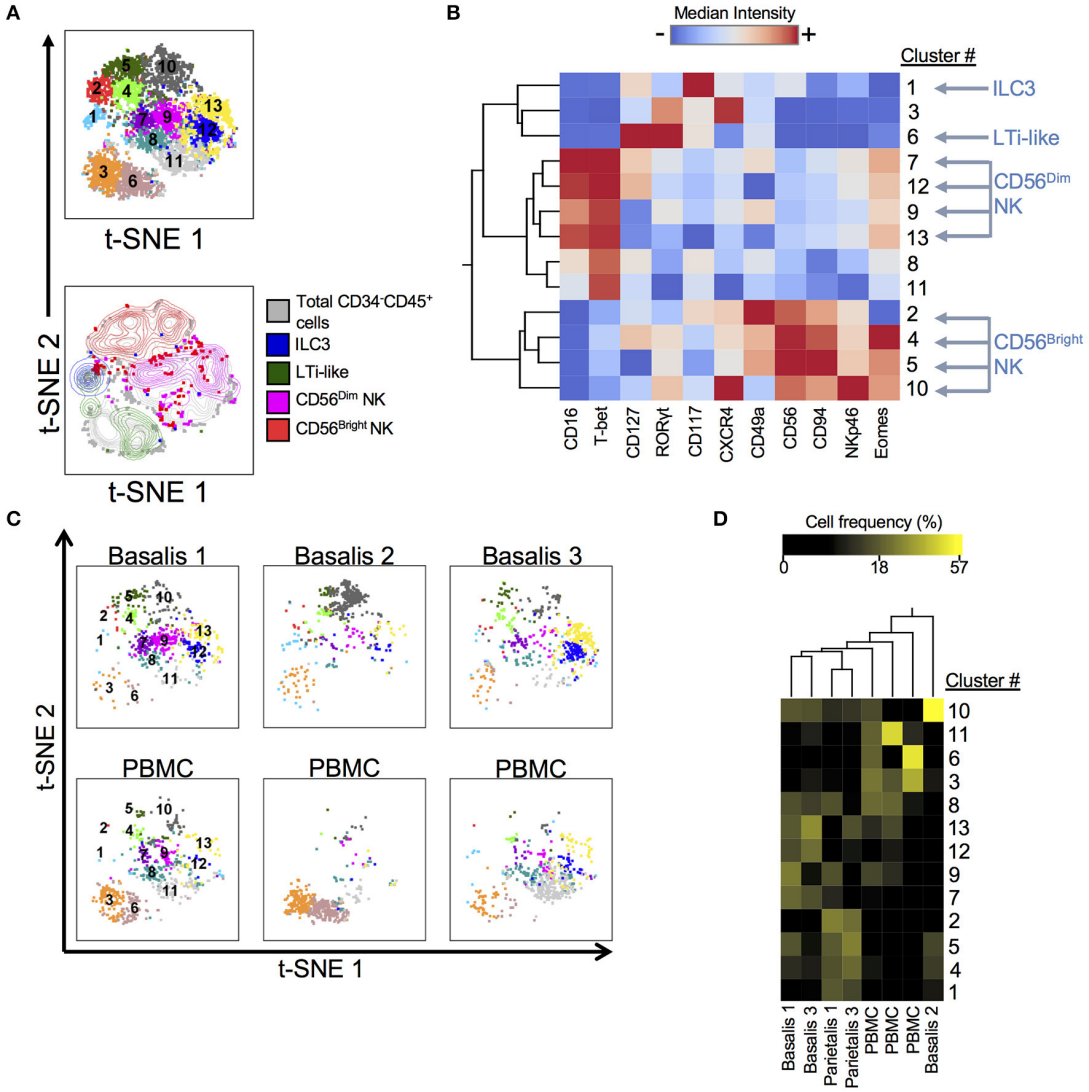
Subset Visualization of Innate Lymphocytes at Human Decidua Basalis. t-SNE was performed on CD34^−^CD45^+^Lin^−^ cells from three experiments. Lin consists of CD3, CD14, and CD19. **(A)** t-SNE map generated from merged data from 3 experiments (top) and indicated cell subsets were manually gated and overlaid onto total CD34^−^CD45^+^Lin^−^ cells (bottom). **(B)** Heatmap shows hierarchical clustering of median surface and intracellular marker expression levels of clusters identified by DensVM. Scale values indicate expression from low (blue) to high (red). **(C)** Separate decidua basalis and PBMC visualized using t-SNE map generated from the merged data set. **(D)** Heatmap shows hierarchical clustering of cluster frequencies within CD34^−^CD45^+^Lin^−^ cells from decidua basalis (D.B), decidua parietalis (D.P), and PBMC (P). Scale values indicate cell frequency from low (black) to high (yellow).

ILCs have recently been identified in early term human decidua (11, 36). We designed and validated a panel that allowed us to identify ILCs, including NK cells, using both surface and intracellular markers at the term maternal-fetal interface (Supplementary Table 2; Supplementary Figure 1). We employed dimensionality reduction (Figure 1), to better visually represent the heterogeneity of ILCs (Lin^−^CD34^−^CD45^+^). DensVM revealed 13 clusters in the merged data set t-SNE map (3 decidua basalis, 2 decidua parietalis, 3 PBMC; Figure 1A, top). CD56^Dim/Bright^ NK cells, ILC3s, and LTi-like cells occupied unique regions of the generated t-SNE map (Figure 1A, bottom). Median fluorescence intensity (MFI) values for each marker were calculated and a heatmap was generated (Figure 1B). In addition, histograms for each marker were generated to better assess the range of expression (Supplementary Figure 2). Individually visualized t-SNE maps showed a tissue specific cluster distribution between decidua basalis and PBMCs (Figure 1C) and clustering of cell frequency confirmed this observation (Figure 1D), with separation of one decidua basalis due to inherent individual variability. Furthermore, low frequencies of ILC1-like cells (clusters 8 and 11; Supplementary Table 4) were seen (Figure 1D) (11). Overall, data reveal overrepresentation of CD56^Bright^ NK cells in decidua when compared to PBMC, with specific enrichment of newly described Eomes^Hi^ NK (C10) and Eomes^Lo^ (C2) cells in decidual tissues (Figure 1D; Supplementary Figure 3).

### RNAseq Reveals Unique Transcriptome of C10 and C2

Differential Eomes expression by C10 and C2 populations tantalized with the possibility that it reflects different cell types, a hypothesis we aimed to test by transcriptome analysis. First, we had to design a panel based solely on surface marker expression that would be able to isolate both clusters (Supplementary Figure 4A). We decided on sorting based on the differential expression of CD49a, with C2 being CD49a^+^ and C10 being CD49a^−^ (strategy in Supplementary Figure 4B). To determine whether this gating strategy was able to recapitulate the differential Eomes expression that we observed through phenotyping, Eomes transcript levels were assessed by qPCR, with higher Eomes transcript levels found in C10 as expected (Supplementary Figure 4C).

**Figure 2:**
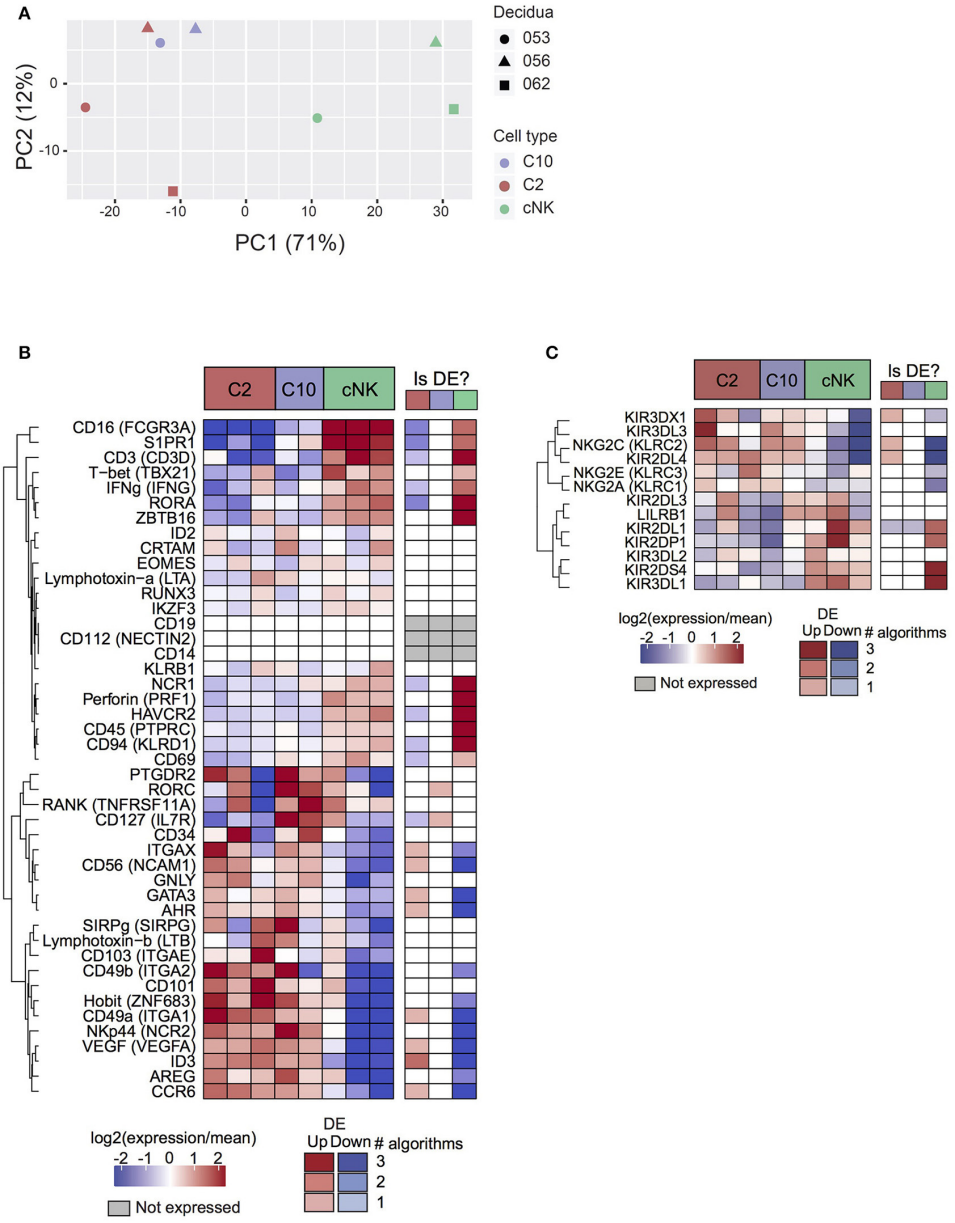
Transcriptome analysis of C10 and C2. C10, C2, and cNKs were sorted and RNA was isolated and sequenced (*n* = 3). **(A)** Principal components analysis of samples by top 500 most varying genes. **(B)** Expression levels of genes of interest (cell sorting panel and canonical ILC genes) from C2, C10, and cNKs. **(C)** Expression levels of KIRs across C2, C10, and cNKs. Whether the gene is called differentially expressed is indicated in the right-hand column, shaded by confidence (number of agreeing DE algorithms). See Data Sheet 2.

Based on principal component analysis (Figure 2A), the first principal component (explaining 71% of variance) separated cNK from C10 and C2. The second principal component (12% of variance) was suggestive of a subtle batch effect by decidua of origin, motivating the inclusion of decidua as a covariate in differential expression analyses. Taken together, PCA analysis indicates that C2 and C10 are highly transcriptionally similar, with cNK being more distantly related.

Surface markers for C2 and C10 corresponded to differentially expressed genes (*Itga1* (CD49a), *Ncam1* (CD56), *Eomes*), validating RNA expression at the protein level (Figures 1B, 2B). Novel ILCs, C10, and C2, exhibited higher expression of *Znf683* (Hobit) and lower expression of *Tbx21* (Tbet), *Zbtb16* (PLZF), *Prf1* Perforin, *Ifng* (IFNγ), *Ncr1* and *Ikzf3* than cNKs. Moreover, C2 expressed additional ILC1 markers *(Itgae, Itgax, Ccr6, Ncr2, Cd101, Sirpg)* (2, 3, 37–39). C2 ILCs, however, also expressed *Id3, Gata3*, and *Ahr*, transcription factors not typically expressed by ILC1s (3, 40, 41). Interestingly, *Rorc* and *Il7r* (CD127) were specifically up-regulated in C10 compared to C2, despite C10 lacking an overall ILC3 phenotype. These trends were corroborated at the protein level, with a lower proportion of T-bet^+^ C10 and C2 ILCs, while both trending toward a higher proportion of RORγt^+^ and GATA-3^+^cells (Supplementary Figure 5). The non-canonical ILC-defining transcription factor expression by C10 and C2 populations, as well as overall transcriptional differences, suggests their divergence from prototypical NK/ILC1 lineage.

Because KIRs are capable of modulating NK cell activity, we determined the variability and level of KIR expression in C10, C2, and cNKs (Figure 2C). KIR expression varied between C10/C2 and cNK, with C10/C2 expressing higher levels of inhibitory KIRs – *KIR3DL3* (binds HLA-C) and *NKG2A* (binds HLA-E) – and activating KIRs – *NKG2C* (binds HLA-E) and *KIR2DL4* (binds HLA-G). CNKs expressed higher levels of activating KIRs, *KIR2DL1* (binds HLA-C) and *KIR2DS4* (binds HLA-C), and inhibitory *KIR3DL1* (binds HLA-B).

**Figure 3:**
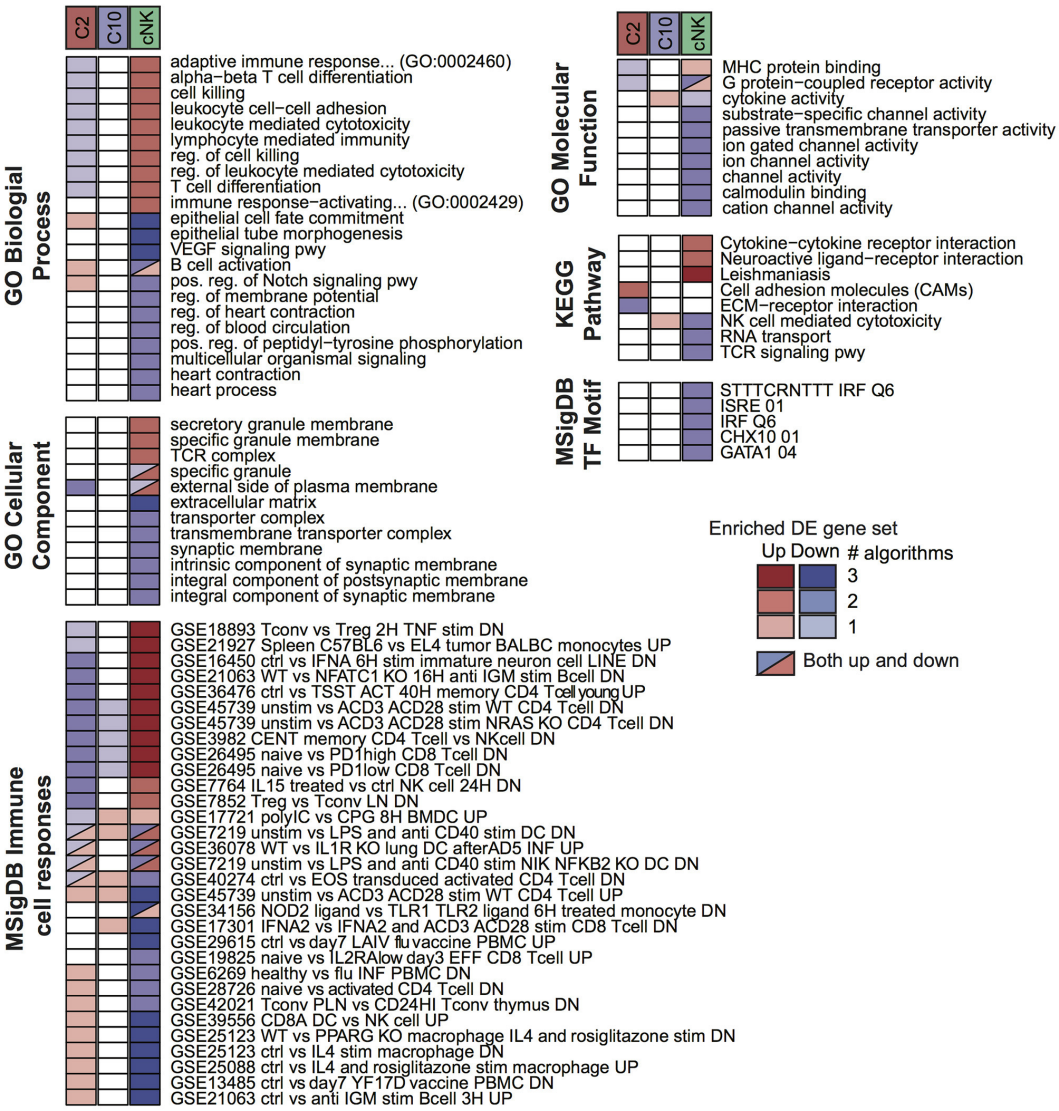
Pathway enrichment analysis of differentially expressed (DE) gene sets. Up- and down-regulated DE gene sets were called for C2, C10, and cNK by comparing each cell type to the other two using three different algorithms (Materials and Methods, “Differential expression analysis”). For each DE gene set and each of six pathway databases from GO, KEGG, and MSigDB, GOSeq was applied to identify pathways that were overrepresented in the DE gene set relative to all annotated genes in the database. Shown are the union of the top 5 terms per gene set and database, filtered to include those identified for at least two cell types or by at least two algorithms. Color indicates the sign of the DE gene set (up/down) and shading indicates the number of DE algorithms for which the pathway was identified.

To probe for pathway-level differences among the cell populations, we tested the differentially expressed gene sets for enrichment with Gene Ontology terms and other curated gene sets (Figure 3). Here, the majority of enriched terms were for genes differentially expressed in cNK vs. both C10 and C2. Genes that were differentially up-regulated in both C10 and C2 compared to cNK were enriched for Gene Ontology biological processes related to angiogenesis, including the VEGF signaling pathway, epithelial tube morphogenesis, and regulation of blood circulation. By contrast, genes that were up-regulated in cNKs compared to the novel ILCs were enriched for terms related to cytotoxicity.

### C2 and C10 Populations Show Similar Expression Profiles to Early Decidual NKs

**Figure 4:**
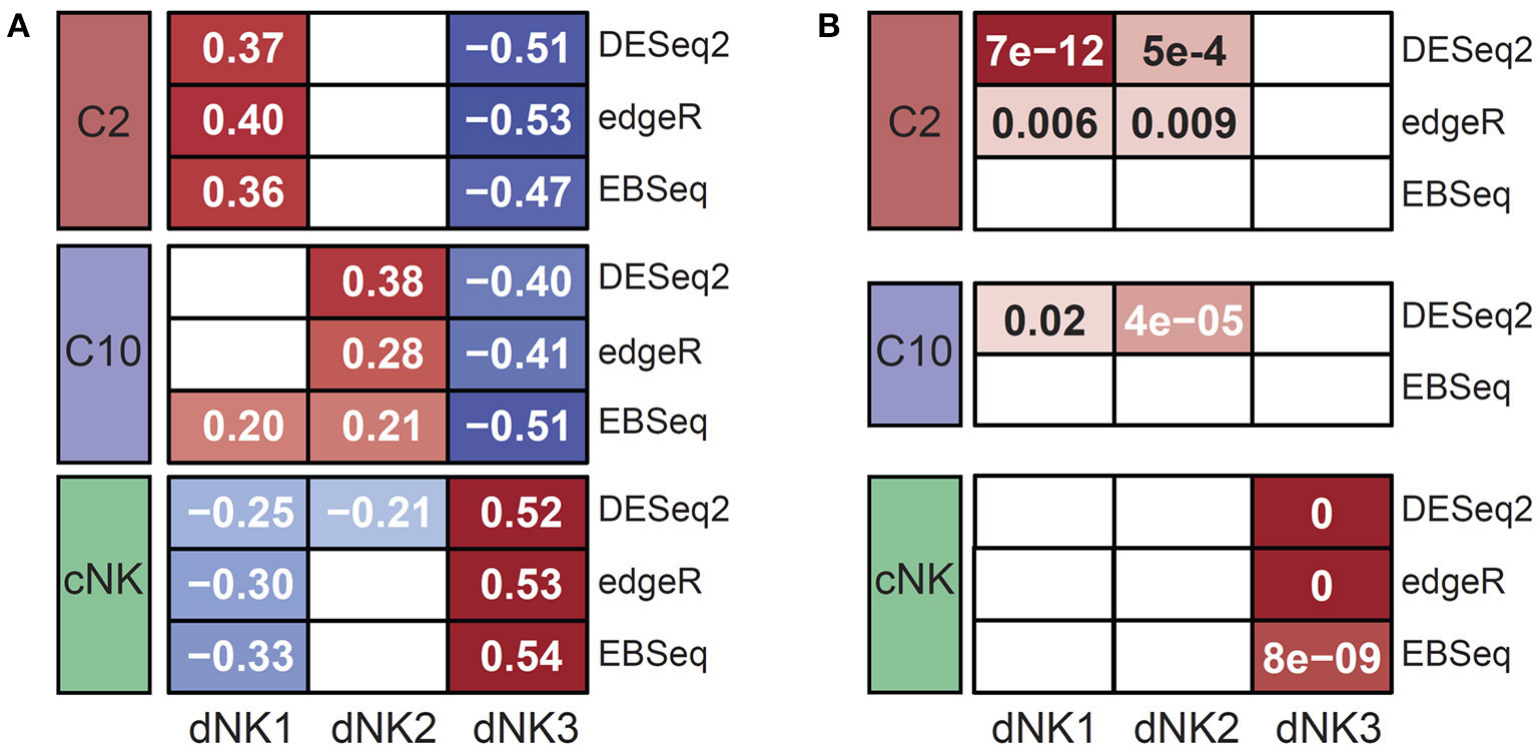
External validation of C10 and C2 by similarity to decidual NKs. Expression levels in C10 and C2 were compared to dNK populations reported by Vento-Tormo et al. (20). **(A)** Correlation of expression of dNK DE genes between dNKs 1-3 and ILC populations C2, C10, cNK. Shaded cells indicate significant correlations; value is Spearman's rank correlation. **(B)** Significance of overlap between up-regulated DE gene sets for dNKs 1-3 and ILCs C10, C2, cNK. Shaded cells indicate significant overlap; values shown are hypergeometric test *p*-values (following Benjamini-Hochberg adjustment for multiple comparisons).

For external validation, gene expression levels were compared to three decidual NK cell populations (dNK1-3) recently defined by scRNA-seq from first trimester decidua (20). Overall, gene expression profiles from C10, C2, and cNK were significantly correlated to differentially expressed genes from dNK populations (Figure 4A), suggesting correspondence between C2 and dNK1, C10 and dNK2, and cNK with dNK3. Up-regulated genes specific to ILC populations C10 and C2 were enriched for marker genes from dNK1-2, whereas up-regulated genes specific to cNKs were enriched for genes specific to dNK3 (Figure 4B). Interestingly, *Vegfa* and *Ifng* were upregulated in C2 and cNK, respectively (Figure 2B) but were not detected in early gestation dNKs. Taken together, these results suggest persistence of pregnancy-specific decidual innate lymphocytes across pregnancy.

### Functional Profile of C10 and C2

**Figure 5:**
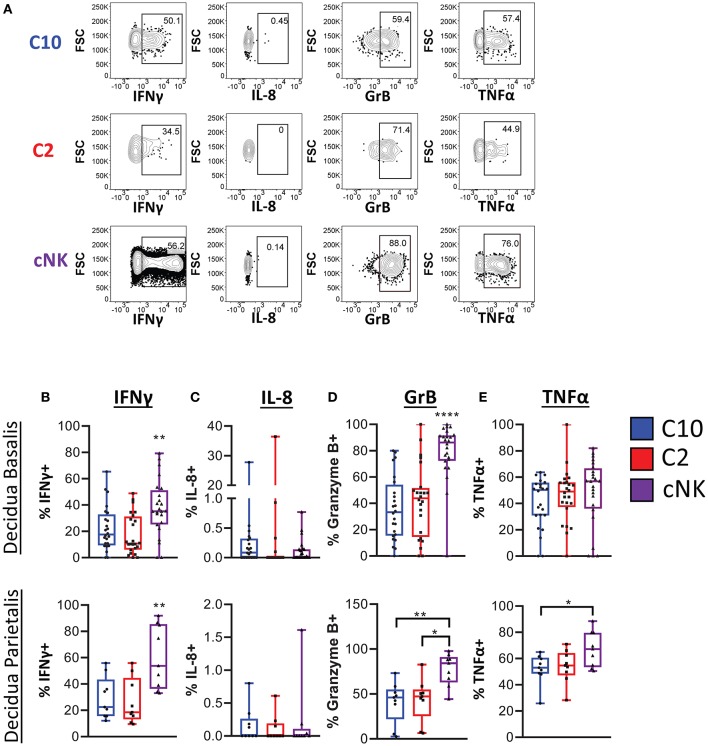
Cytokine production by novel ILCs. C10 and C2 skew towards NK-type cytokines. Lineage Negative (CD3, CD14, CD19), CD34^−^CD45^+^ cells were activated with PMA/Ionomycin for 6 h. Cytokine production was assessed via intracellular labeling. **(A)** Representative gating scheme of CD56^Bright^ CD94^+^CD16^−^CD127^−^CD49a^−^ (C10), CD49^+^ (C2), and of CD56^Dim^CD16^+^CD127^−^CD117^−^ from the decidua basalis showing NK type cytokine production **(B–E)** Quantification of NK type cytokine production by C10, C2, and cNK. Decidua basalis, *n* = 24; decidua parietalis, *n* = 9. Data represented as max/min, median, and 25 and 75th percentiles. Statistical significance was determined by ANOVA, followed by Tukey *post-hoc* tests. *p < 0.05, **p < 0.005, ****p < 0.00001. For statistical details see Supplementary Table 5. Data is provided in Data Sheet 1.

We next determined functional potential of C10 and C2 populations. A screen of various chemokines and cytokine relevant to the decidua showed that IP-10, CCL5, CCL3, TNFα, IFNγ, IL-4, IL-2, as well as CCL4 were produced by both C10 and C2 after PMA/Ionomycin activation (Supplementary Figure 6). A similar expression profile was obtained in cNKs from technical control PBMCs (Supplementary Figure 6). Because NK- and T_H_17 cytokines have been associated with tissue remodeling (9, 42–44), we investigated whether C10 and C2 produce them (Figure 5A; Supplementary Figure 7). IFNγ, one of the primary cytokines produced by NK cells (45–47), is associated with spiral artery remodeling and angiogenesis in early pregnancy (9, 42, 48). After PMA/Ionomycin treatment, we found that decidual cNKs had a higher proportion of IFNγ-positive cells and higher iMFI value (Supplementary Figure 7A), indicative of total functional response, compared to decidual C10 and C2 ILCs (Figure 5B) despite all having the same tissue of origin, suggesting that decidual C10 and C2 ILCs are primed for lower IFNγ output.

IL-8, implicated in stimulating migration of extravillious trophoblasts (49), was produced by a very small proportion of C10, C2, and cNKs from both the decidua basalis and parietalis, with no difference across all cell types (Figure 5C). This suggests that term decidual C10 and C2 might be more involved in angiogenesis, orchestrating endothelial cell remodeling, as opposed to directing trophoblast migration, while recognizing that these functional modules may be pregnancy-stage specific.

Granzyme B is one of many cytotoxic factors produced by NK cells (50). Both C10 and C2 ILCs had a lower frequency of Granzyme B-positive cells compared to cNKs upon PMA/Ionomycin activation (Figure 5D). Activation with PMA/Ionomycin also resulted in a lower iMFI value in C10 and C2 ILCs, suggestive of lower Granzyme B production compared to cNKs (Supplementary Figure 7B).

TNFα, another pro-inflammatory cytokine that is produced by NK cells (45, 51), has been associated with pregnancy loss (52, 53). We found that, while there was no difference in the proportion of TNFα-positive ILC subsets from the decidua basalis, cNKs from the decidua parietalis did have a higher frequency of TNFα-positive cells to C10 and C2 ILCs (Figure 5E). Interestingly, as indicated by iMFI, the total TNFα functional response was not different across ILC subsets (Supplementary Figure 7C). Thus, although TNFα might be detrimental to the pregnancy, dILCs are primed for its production given the right stimulus.

**Figure 6:**
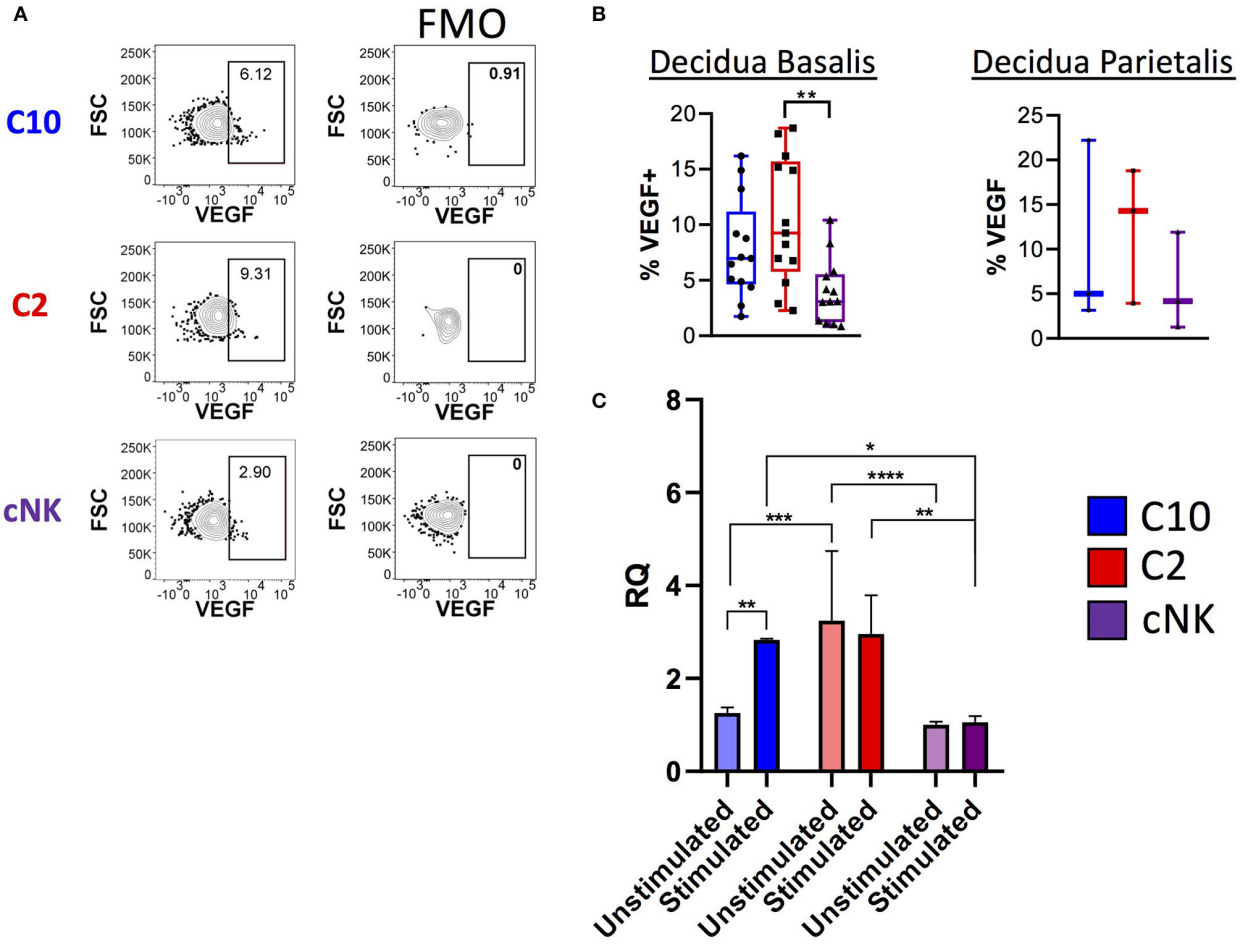
Production of VEGF by novel ILCs C10 and C2. Lineage Negative (CD3, CD14, CD19), CD34^−^CD45^+^ cells were activated with PMA/Ionomycin for 6 h. **(A)** Left, Representative gating scheme of C10, C2, and cNK showing VEGF production. Right, Fluorescence minus one control for VEGF staining. **(B)** Quantification of VEGF production by C10, C2, and cNK. Decidua basalis, *n* = 13; decidua parietalis, *n* = 3. Data represented as max/min, median, and 25 and 75th percentiles. Statistical significance was determined by ANOVA, followed by Tukey *post-hoc* tests. **p < 0.005. **(C)** Sorted C10, C2, and cNKs cells from the decidua were stimulated with cytokines (IL-12/IL-15/IL-18) for 24 h, then processed for RNA extraction, followed by qPCR analysis. Data represented as mean ± SEM Statistical significance was determined by 2- way ANOVA, followed by Tukey *post-hoc* tests. *p < 0.05, **p < 0.005, ***p < 0.0005, ****p < 0.00001. Unstimulated, *n* = 4; stimulated, *n* = 2. For statistical details see Supplementary Tables 7, 8. Data is provided in Data Sheet 1.

Because transcriptome analysis revealed upregulation of *Vegf* and VEGF signaling pathways in C2 and C10 (Figures 2B, 3), we asked whether VEGF is produced by decidual C10 and C2 ILCs (Figure 6; Supplementary Figure 8). Activation with PMA/Ionomycin resulted in a higher proportion of VEGF^+^ C2 ILCs compared to cNKs (Figure 6B, left) and higher levels of secreted VEGF by C2 (Supplementary Figure 8B). However, due to limits in tissue availability, we were not able to determine if the same pattern applies to decidua parietalis (Figure 6B, right). Quantification of *Vegfa* transcript also supports our observation of higher VEGF protein levels in C2 ILCs (Figure 6C). Interestingly, when comparing across tissues, we found that decidual C10 and C2 had a higher proportion of VEGF^+^ cells compared to their PBMC counterparts (Supplementary Figure 8A).

We then asked whether these novel ILCs, and especially RORγt-expressing C10 population are capable of producing ILC3/T_H_17-type cytokines (IL-22 and IL-17A). After activation with PMA/Ionomycin, the production of IL-22 and IL-17A were assessed (Supplementary Figures 6, 9). The proportion of IL-22 and IL-17A positive cells within C10, C2 and cNK were low across all tissues (Supplementary Figure 9). Furthermore, we found that neither C10 nor C2 ILCs had secreted levels of IL-17A above the Minimum Detectable Concentration (MDC) (3.4 pg/mL; Supplementary Figure 6). Overall, these results suggest that, although similar to cNKs, C10 and C2 are functionally more similar to each other, do not demonstrate differential RORγt-driven IL-22 or IL-17A production, and have the capacity to produce NK-type cytokines, as well as VEGF.

### C10 and C2 Response to Cytokine Stimulation

**Figure 7:**
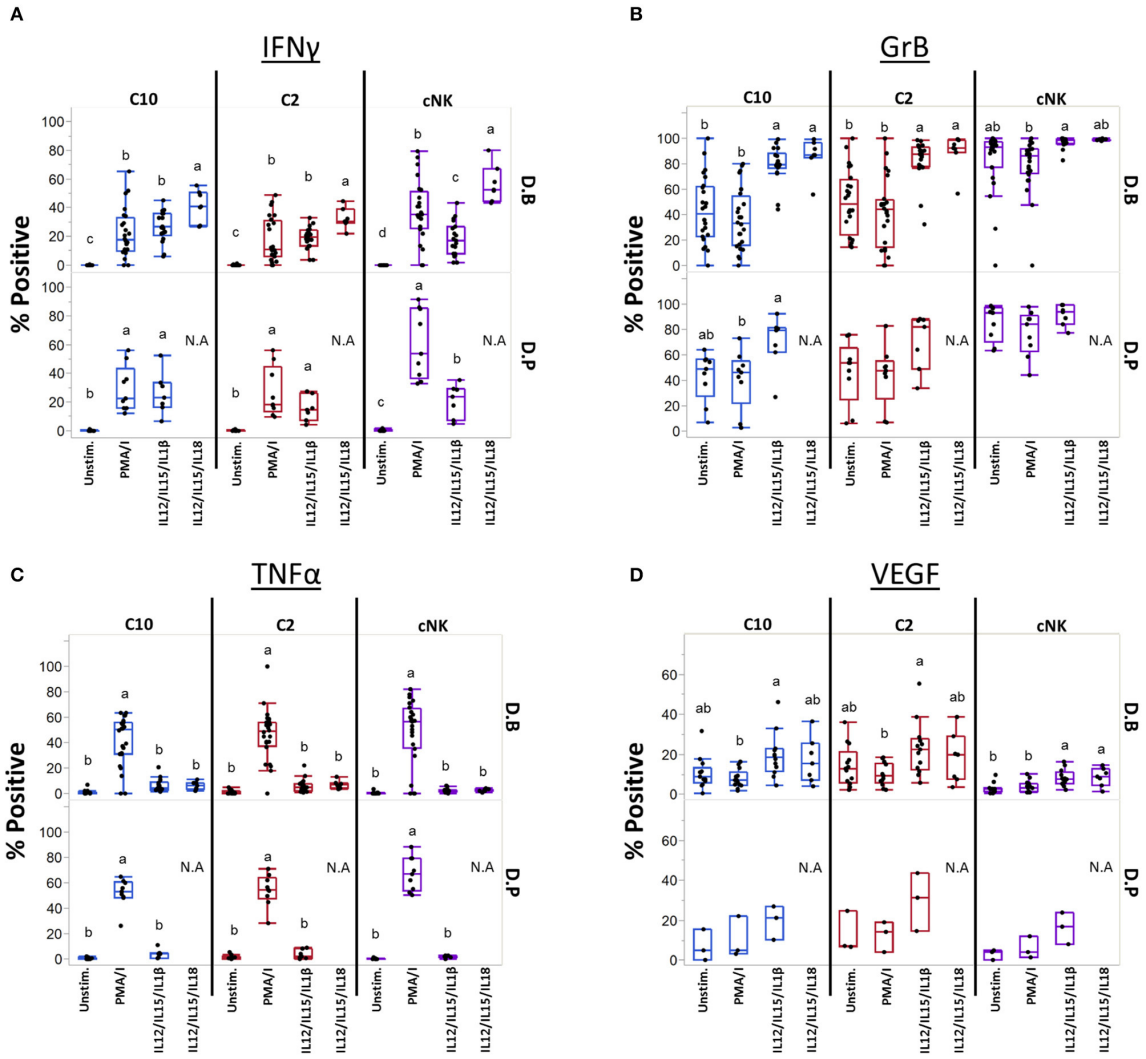
Unique ILCs respond to cytokine stimulation. C10, C2, and cNKs were stimulated with PMA/Ionomycin, IL-12/IL-15/IL-1β, or IL-12/IL-15/IL-18 and production of **(A)** INFγ, **(B)** Granzyme B, **(C)** TNFα, and **(D)** VEGF was assessed. Data represented as max/min, median, and 25 and 75th percentiles. Statistical significance was determined by ANOVA followed by Tukey test and are demonstrated by letters, with different letters indicating statistical differences within a subset (<0.05). Additionally, comparisons across subsets within tissues were performed, for simplicity *p*-values are found in Supplementary Table 10. For (**A–C**, unstim and PMA/Ionomycin): Decidua basalis, *n* = 24; decidua parietalis, *n* = 9. For (**A–C**, IL12/IL15/IL1β): Decidua basalis, *n* = 19; decidua parietalis, *n* = 7 (**A–C**, IL12/IL15/IL18): Decidua basalis, *n* = 7. For (**D**, unstim, PMA/Ionomycin, IL12/IL15/IL1β): Decidua basalis, *n* = 13; decidua parietalis, *n* = 3. For (**D**, IL12/IL15/IL18): Decidua basalis, *n* = 7. N.A = not analyzed. For statistical details see Supplementary Table 10. Data is provided in Data Sheet 1.

Cytokines IL-12, IL-15, IL-18, and IL-1β are produced at the maternal-fetal interface and activate NK cells (54–56), leading us to ask if they can similarly influence C10 and C2 cells (Figure 7). Production of IFNγ was induced by both cytokine treatments in C10, C2, and cNK from both the decidua basalis and parietalis, with IL-12/IL-15/IL-1β leading to a lower frequency of IFNγ-positive cNKs (Figure 7A). Interestingly, cNKs had a higher proportion of IFNγ-positive cells upon IL-12/IL-15/IL-18 treatment compared to C2 ILCs (Figure 7A; Supplementary Table 10), however, C10 ILCs had an overall higher IFNγ functional response (Supplementary Figure 10). Due to limits in tissue recovery, we were unable to detect a similar pattern in decidua parietalis ILCs.

Next, we assessed Granzyme B production after activation with cytokines (Figure 7B). First, cNKs had higher basal levels of Granzyme B^+^ cells compared to C10 and C2 (Figure 7B; Supplementary Table 10). Despite cytokine treatments leading to higher proportions of Granzyme B^+^ C10 and C2 cells (Figure 7B), cNKs maintained a higher functional response across treatments (Supplementary Figure 10).

Unlike what we observed in IFNγ and Granzyme B after cytokine activation, TNFα production was not induced by IL-12/IL-15 in combination with IL-1β or IL-18 activation, with TNFα^+^ proportions reaching only the unstimulated levels in all ILC subsets (Figure 7C). Furthermore, there were no differences between ILC subsets within any of the treatments (Figure 7C; Supplementary Figure 10; Supplementary Table 10).

We asked whether cytokine stimulation would lead to VEGF production (Figure 7D; Supplementary Figures 10, 11). Within C10 ILCs from the decidua basalis, we found that IL-12/IL-15/IL-1β activation led to a higher proportion of VEGF^+^ cells compared to PMA/Ionomycin activation. A similar pattern was observed in C2 ILCs of the decidua basalis, as activation with IL-12/IL-15/IL-1β led to a higher percentage of VEGF^+^ cells compared to PMA/Ionomycin activation, however, not when compared to the unstimulated. Interestingly, the unstimulated C2 had a higher proportion of VEGF^+^ cells compared to unstimulated cNKs, with IL-12/IL-15/IL-1β stimulation leading to higher VEGF^+^ cells in both C10 and C2 subsets compared to cNKs (Figure 7D; Supplementary Table 10). We also noted that under IL-12/IL-15/IL-1β treatment both C10 and C2 ILCs from the decidua basalis had a higher iMFI compared to cNKs (Supplementary Figure 10).

Lastly, we activated C2 and C10 with IL-23 and IL-1β, both known to activate ILC3s. We found that stimulation with IL-23, either alone or in combination with PMA/Ionomycin or IL-1β, did not lead to the production of IL-17A or IL-22 (Supplementary Figure 12).

### Polyfunctional Properties of Novel Decidual ILCs

**Figure 8:**
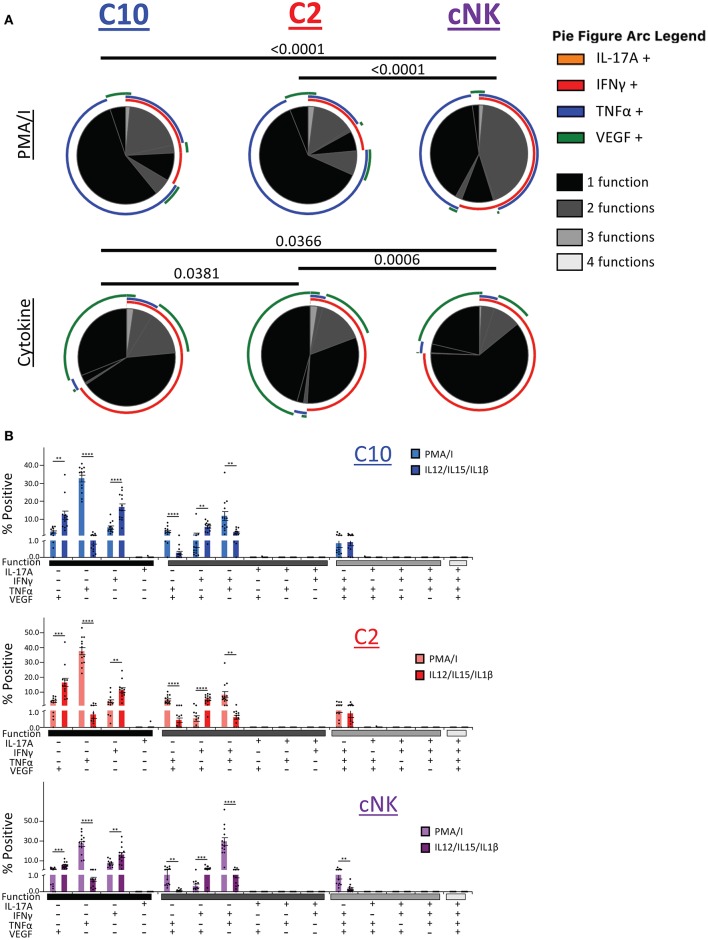
Polyfunctional properties of Novel ILCs. Novel ILCs C10 and C2 display unique polyfunction properties upon PMA/Ionomycin or cytokine (IL-12/IL-15/IL-1β) activation. **(A)** Pie charts show the proportion of functions that C10, C2, and cNK display after activation with PMA/Ionomycin (top) and cytokines (bottom). Arcs indicate the proportion that produce indicated factor IL-17A, INFγ, TNFα, VEGF. **(B)** Proportion of positive cells for given combinations of assessed factors after PMA/Ionomyin or cytokine activation. *n* = 13. Data represented as mean ± SEM. Statistical significance was determined by Student's *t*-test. **p < 0.005, ***p < 0.0005, ****p < 0.00001. For statistical details see Supplementary Table 12. Data is provided in Data Sheet 1.

We next asked whether C10, C2, and cNKs are able to secrete multiple cytokines and growth factors simultaneously upon activation (Figure 8). Because we were limited in the number of markers we could simultaneously label, we divided our functional stains into two panels consisting of IL-17A, IFNγ, TNFα, and VEGF or Granzyme B, IL-8, IL-17A, and TNFα. First, we found that the majority of C10 ILCs in the decidua were monofunctional (70.3%), followed by bifunctional (28.3%), with very few trifunctional cells (1.5%) upon PMA/Ionomyin activation. C2 ILCs followed a very similar pattern, with most being monofunctional (74.9%), a lower proportion of bifunctional (23.0%) and even fewer trifunctional (2.1%) cells (Figure 8A, top). On the other hand, cNKs were evenly split between monofunctional (52.5%) and bifunctional (46.0%), with very few trifunctional (1.5%) cells (Figure 8A). The functional outputs (i.e., factors produced) were also different between C10/C2 and cNK as indicated by Monte Carlo simulation [partial permutation (33)] analysis (Figure 8A, top).

Upon cytokine (IL-12/IL-15/IL-1β) stimulation, functional distribution remained similar in C10 (75.8, 21.9, and 2.3%) and C2 (79.5, 18.1, and 2.4%). However, cNK functional distribution shifted, with an increase in monofunctional cells (85.6, 13.7, and 0.7%) (Figure 8A, bottom). Similar to the PMA/Ionomycin treated group, we detected functional differences between C10/C2 and cNK, however, differences between C10 and C2 themselves were revealed by cytokine stimulation (Figure 8A, bottom).

We further investigated the differences in functional properties induced by cytokine stimulation compared to PMA/Ionomycin stimulation (Figure 8B). In all three decidual ILC subsets (C10, C2, and cNK), cytokine treatment led to an increase in VEGF^+^ and IFNγ^+^ monofunctional cells, with a decrease in TNFα^+^ monofunctional cells. This corresponds with a decrease in TNFα^+^VEGF^+^ and IFNγ^+^TNFα^+^ bifunctional cells, accompanied with an increase in IFNγ^+^VEGF^+^ bifunctional cells. Interestingly, cNKs had a decrease in trifunctional IFNγ^+^TNFα^+^VEGF^+^ cells (Figure 8B).

**Figure 9:**
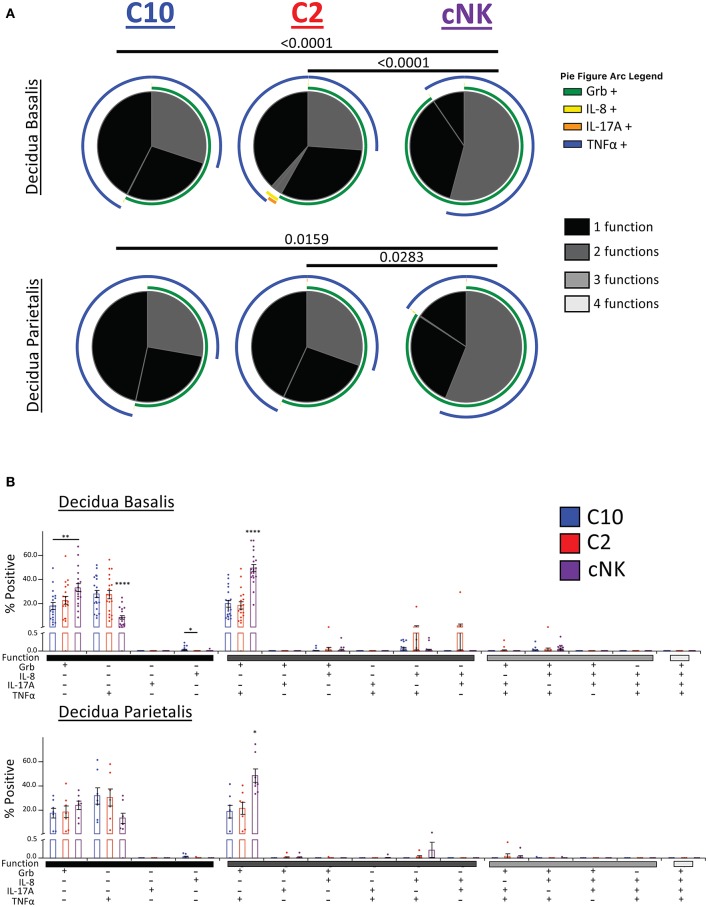
Activation of C10 and C2 ILCs with PMA/Ionomycin Reveals Preference for TNFα. Activation of C10 and C2 led to a higher proportion of TNFα^+^ cells compared to cNKs. Decidua basalis, *n* = 19; decidua parietalis, *n* = 7. Data represented as mean ± SEM. Statistical significance was determined by ANOVA followed by Tukey test. *p < 0.05, **p < 0.005, ****p < 0.0001. For statistical details see Supplementary Table 13. Data is provided in Data Sheet 1.

Our second functional panel revealed that the majority of C10 ILCs in the decidua basalis were mostly monofunctional (69.78%), followed by bifunctional (30.18%), with a negligible amount of trifunctional cells (0.04%). Similarly, the majority of C2 ILCs were monofunctional (70.34%), followed by bifunctional (29.55%), and a very small percentage of trifunctional cells (0.10%). Decidua basalis cNKs were split almost evenly between monofunctional (45.80%) and bifunctional (54.13%) cells, with a very small percentage of trifunctional cells (0.07%) (Figure 9A, top). ILCs from the decidua parietalis followed a similar pattern as that see in the decidua basalis, with the majority of C10 and C2 being monofunctional (72.25 and 69.48%, respectively). Similar, cNK from the decidua parietalis were closely evenly split between monofunctional (43.61%) and bifunctional (56.33%) (Figure 9A, bottom).

The monofunctional distribution of C10 was consistent with our earlier results (Figure 5D), except for Grb^+^ monofunctional cells from the decidua parietalis (Figure 9B). A slightly higher percentage of C10 ILCs from the decidua basalis were IL-8^+^ compared to C2 ILCs. Furthermore, a higher proportion of cNKs in both the decidua basalis and parietalis were GrB^+^TNFα^+^ compared to C10/C2 (Figure 9B).

## Discussion

Pregnancy requires collaboration between maternal immune cells and semi-allogeneic fetal cells. Development of pregnancy related diseases, such as preeclampsia and intrauterine grown restriction (IUGR), are often attributed to dysregulation of this collaborative system. The maternal-fetal interface, although restricted in its immune composition, maintains a population of specialized decidual natural killer cells (dNKs), defined by a CD56^Bright^CD16^−^ phenotype. Although dNKs have been implicated in the early implantation process (9, 57), their link to pathology has been inconsistent (16, 17). ILCs have emerged as important players in mucosal defense and homeostasis, and have been identified in both human and mouse decidua (11, 36, 58). Here we present previously underappreciated heterogeneity within decidual CD56^Bright^CD16^−^ ILCs and the identification of two novel subsets, C10 and C2. Globally, transcriptome analysis showed that C10 and C2 are different from cNKs, and each other, despite all being of decidual origin. Furthermore, we show that these novel ILCs are polyfunctional with specific profile being dependent on the nature of the stimulus.

We first validated our panels for the identification of ILCs in the term human decidua and confirmed the presence of dNKs and ILC3s (9, 11, 59). Our application of tSNE/DensVM highlighted the diversity of CD56^Bright^CD16^−^ ILCs in the decidua. Cellular clustering also delineated a specific ILC signature for term human decidua, a pattern that we had previously observed in the T and dendritic cell compartments (18). More importantly, the heterogeneity within the CD56^Bright^ population suggests the presence of multiple ILCs that play redundant or conflicting roles in the decidua, thus revealing a possible explanation for the discord between prior studies attempting to link dNK number and function with clinical outcomes (16, 17).

ILC1s are characterized by the expression of CD49a, CD103, NKp44, and T-bet (3, 37, 38). Our data suggests that C2 has some ILC1 characteristics, including the expression of CD49a, low expression of perforin and S1PR1 (37, 38) (Figure 2B). However, C2 decidual ILCs do not express T-bet or Runx3, transcription factors expressed by ILC1s (2, 47). In the mouse, however, a uterine ILC1 group has been identified with low expression of T-bet (38), suggesting that C2 ILCs might represent a human uterine ILC1 group, although Runx3 expression in these cells was not explored. GATA-3 expression would suggest that C2 are decidual ILC2s, which have recently been identified in human decidua (58), however, the expression of CD56 by C2 would indicate that they are not ILC2s. Interestingly, the expression of CD103 (*Itgae*) by dNKs has been noted in multiple studies (60–63) supporting the idea that dNKs is a highly diverse group. Moreover, despite expression of Eomes, C10 lack expression of CD49a, diverging from the previously defined decidual NK cell (12).

Regulatory ILCs (ILCregs), that express ID3 and secrete IL-10, have recently been identified both in mice and in humans (41, 64, 65). Interestingly, both C10 and C2 express ID3 suggesting overlapping features with ILCregs. However, we found that under PMA/Ionomycin treatment, C10 and C2 ILCs did not produce IL-10 and their response to TGFβ remains unknown (41, 64, 65). Moreover, C2 also express aryl hydrocarbon receptor (*Ahr*) and *Gata3*, which are associated with ILC development (65). This, together with their production of IFNγ, TNFα, and Granzyme B suggests that C2 have an ILC1-like phenotype, despite their low T-bet expression. Even though C10 displays an ILC3-like phenotype (expression of RORγt and CD127), we found that C10 were not responsive to IL-23 or IL-1β, suggesting that they might represent a separate ILC group. The possibility remains that C10 represents an intermediate between ILC3-ILC1 transition. However, C10 was found not to express Aiolos (*Ikzf3*), a transcription factor, that together with T-bet, is important in ILC3 to ILC1/NK transition (66, 67), although tissue-specific requirements for this process, if present in the decidua, are as yet unknown.

Functional analysis of C10 and C2 did not reveal profound differences in cytokine response to stimulation, suggesting a level of redundancy in the system. Overall, both clusters had a lower proportion of IFNγ positive cells compared to cNKs as expected. Although IFNγ has been shown to be necessary for early pregnancy remodeling (42), high levels of IFNγ have also been associated with pregnancy complications (68). This suggests that decidual C10 and C2 are programmed by the pregnant environment to produce the optimal level of IFNγ. Interestingly, dNKs have been shown to retain memory of pregnancy, priming them for future pregnancies (48). Although we do not have data on parity, our results suggest that these novel ILCs retain an imprint of their native pregnant environment as the proportion of VEGF-positive cells in unstimulated decidual C10 and C2 was higher compared to PBMC counterparts (Supplementary Figure 8A).

Activation with cytokines, a more physiological stimulus, revealed that C10 and C2 skewed their polyfunctional profile. Overall, we found that both C10 and C2 shifted toward IFNγ/VEGF production, away from TNFα production. Indeed, we saw a dramatic decrease in the production of TNFα upon cytokine treatment, with a more pronounced decrease in decidual C10 and C2 ILCs. This suggests that decidual ILCs are capable of producing TNFα, as indicated by the PMA/Ionomycin treatment, but do not produce much under physiological conditions. In fact, TNFα has also been associated with pregnancy complications (52) and, although, small amounts of TNFα are sustainable in pregnancy, high levels can have detrimental outcomes. Production of VEGF by C2/C10 could mediate trophoblasts migration in late gestation (69). However, the primary role of C10 and C2 might be to maintain steady levels of IFNγ, as IFNγ plays a role in maintaining tissue homeostasis (70) and preventing further trophoblast invasion (69, 71). In addition, IFNγ can suppress production of matrix metalloproteinases (72, 73), which have been associated with labor (74, 75). This suggests the C10 and C2 might be involved in maintaining pregnancy by suppressing effector molecules involved in the parturition process. Interestingly, we did not observe any major functional differences between decidual basalis and parietalis C10 and C2 ILCs (data not shown) even though the decidual basalis ILCs are derived from the implantation/invasion site itself, suggesting that soluble/hormonal factors might be more important in programming cytokine production by decidual ILCs.

Lastly, our RNAseq data shows concordance between early term decidual ILCs and late term novel decidual ILC subsets (C10 and C2) and cNKs described here, suggesting a continuity of these cells types from early pregnancy through late gestation. It remains to be determined whether the novel decidual ILC subsets identified here have a similar function to those identified in early term decidua. Nevertheless, these findings validate the use of term decidua as a reasonable cellular source for studies of human decidual innate lymphocytes across gestation.

## Data Availability Statement

RNA sequencing data that support the findings of this study have been deposited in GEO with the primary accession codes GSE129555 (https://www.ncbi.nlm.nih.gov/geo/query/acc.cgi?acc=GSE129555). All other data that support the findings of this study are available in the source data file (Data Sheets 1, 2) or from the corresponding author upon reasonable request.

## Ethics Statement

De-identified term human (>37 wks GA) placental samples were collected from normal elective cesarean sections under the UnityPoint Health—Meriter IRB protocol (#2017-004) and UW Obstetrical Tissue Bank IRB protocol (#2014-1223). As per IRB review at UnityPoint Health—Meriter, these collections did not constitute Human Subjects Research, but given sensitive nature of perinatal care were fully reviewed to achieve that determination.

## Author Contributions

JV and AS designed research. CT, JV, and GL acquired tissue samples. JV performed experiments. JV, DC, IO, AS analyzed data. JV, DC, IO, and AS wrote manuscript. AS supervised the project.

### Conflict of Interest

The authors declare that the research was conducted in the absence of any commercial or financial relationships that could be construed as a potential conflict of interest.
