# Supplementary material for: Transcriptional and Functional Programming of Decidual Innate Lymphoid Cells
Source: Front Immunol. 2020 Jan 24;10:3065. doi: 10.3389/fimmu.2019.03065 (PMC6992589; doi:10.3389/fimmu.2019.03065)
Supplement: Supplementary file 3 [file Data_Sheet_3.docx]

Supplementary Material

Transcriptional and Functional Programming of Decidual Innate Lymphoid Cells

Jessica Vazquez^1^, Deborah Chasman^1,4^, Gladys Lopez^1^, Chanel T. Tyler^1,2^, Irene M Ong ^1,4,5,^ Aleksandar K. Stanic^1,3^

^1^Division of Reproductive Sciences, Department of Obstetrics and Gynecology, University of Wisconsin-Madison, Madison, WI, USA

^2^Division of Maternal-Fetal Medicine, Department of Obstetrics and Gynecology, University of Wisconsin-Madison, Madison, WI, USA

^3^Division of Reproductive Endocrinology and Infertility, Department of Obstetrics and Gynecology, University of Wisconsin-Madison, Madison, WI, USA

^4^Department of Biostatistics and Medical Informatics, University of Wisconsin-Madison, Madison, WI, USA

^5^Carbone Comprehensive Cancer Center, University of Wisconsin-Madison, Madison, WI, USA

*** Correspondence:**Aleksandar K. Stanic
stanickostic@wisc.edu

##
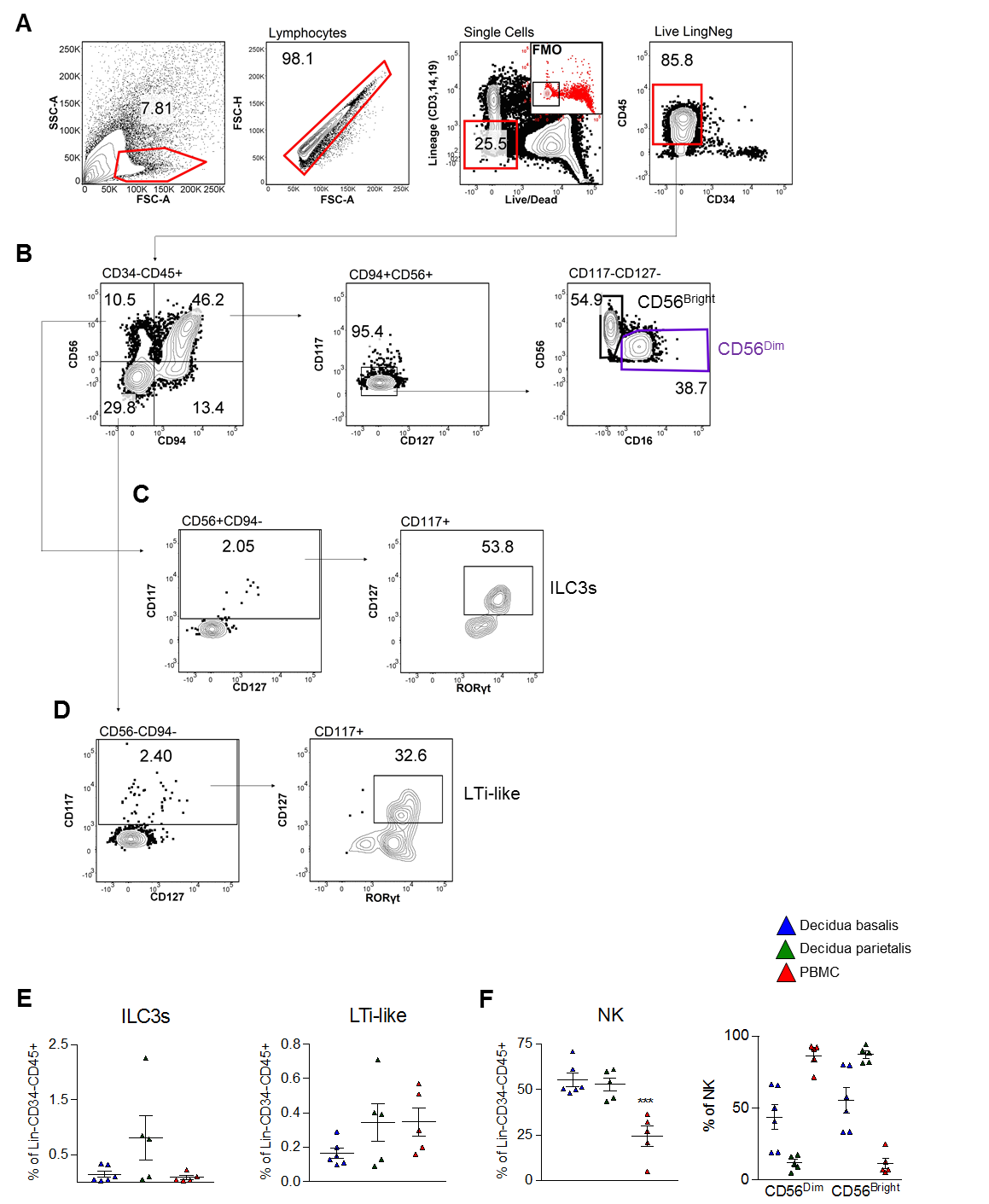
Supplementary Figure 1. Innate lymphoid cells identified in human term decidua. (A) Gating scheme identifying lineage negative lymphocytes. Lineage consists of CD3, CD14, and CD19. (B) Identification of NK cells and CD56^Dim^ and CD56^Bright^ NK subsets. (C) Gating scheme identifying RORyt ILC3 and (D) LTi-like cells. Data representative of 6 experiments. (E) Percentage of ILC3s (left) and LTi-like cells (right). (F) Percentage of NK (left) and NK substypes (right), CD56^Dim^ and CD56^Bright^. Decidua Basalis (n = 6), Decidua Parietalis (n = 5), and PBMCs (n = 5). All data presented as mean ± SEM. Statistical significance was determined by ANOVA followed by post-hoc Tukey analysis. ***p < 0.0005.


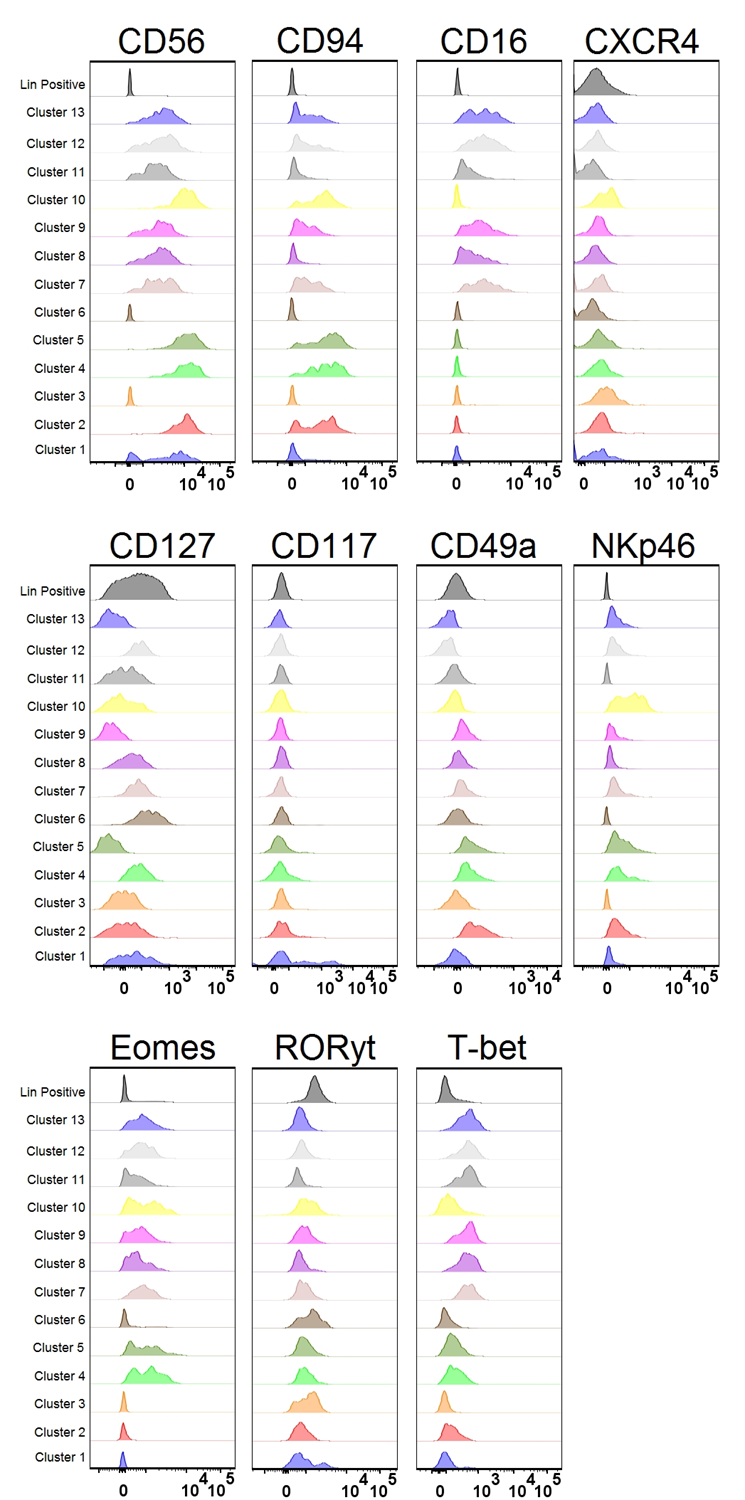


**Supplementary Figure 2.** Histograms displaying the range of expression for the markers assessed by tSNE/DensVM for each cluster identified and for Lineage Positive (CD3, CD14, CD19). Related to Figure 1B.


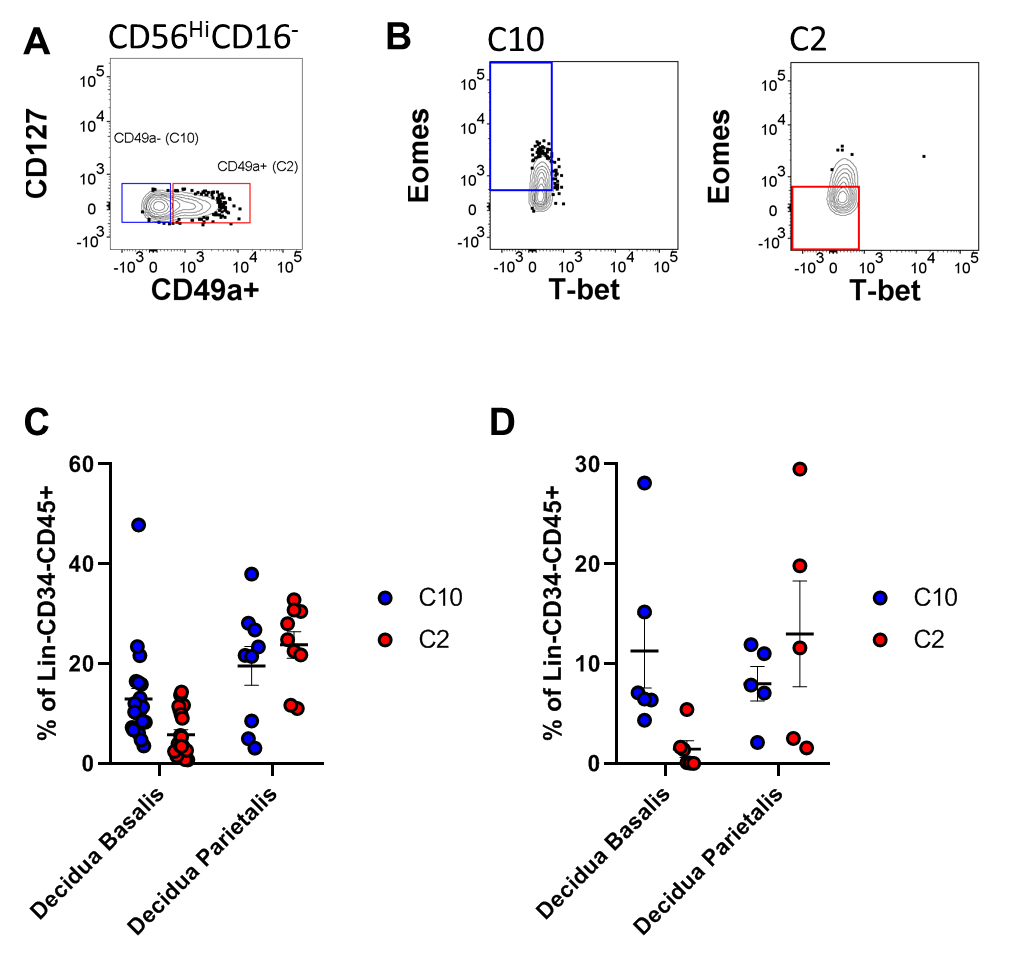


**Supplementary Figure 3.** Proportions of C10 and C2 decidual ILCs. Identification of CD49a^-^ (C10) and CD49a^+^ (C2) by **(A)** surface marker expression or **(B)** transcription factor expression in the decidua basalis. Proportion of C10 and C2 from total Lineage negative cells based on **(C)** surface phenotype or **(D)** transcription factor expression. For C, Decidua Basalis (n = 11), Decidua Parietalis (n = 7); For D, Decidua Basalis (n = 6), Decidua Parietalis (n = 5). All data presented as mean ± SEM.


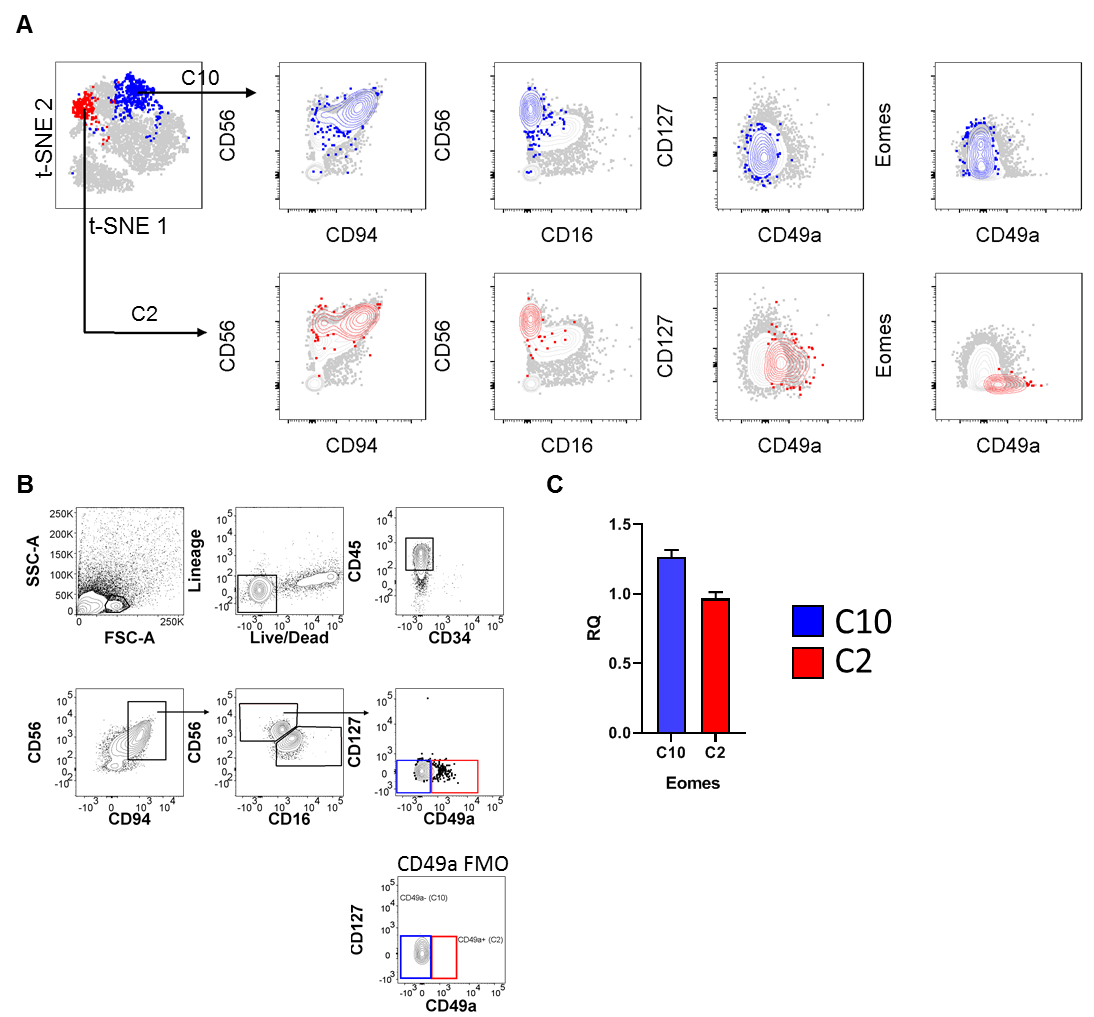


**Supplementary Figure 4.** Gating scheme for the isolation of C10 and C2 dILCs. **(A)** Expression of surface markers of clusters #2 and #10 used to determine proper sorting gating scheme. **(B)** Sorting gating scheme designed to isolate clusters #2 and #10 dILCs **(C)** Relative quantification (RQ) of Eomes transcript expression by qPCR in sorted C10 and C2 dILCs (n = 2).


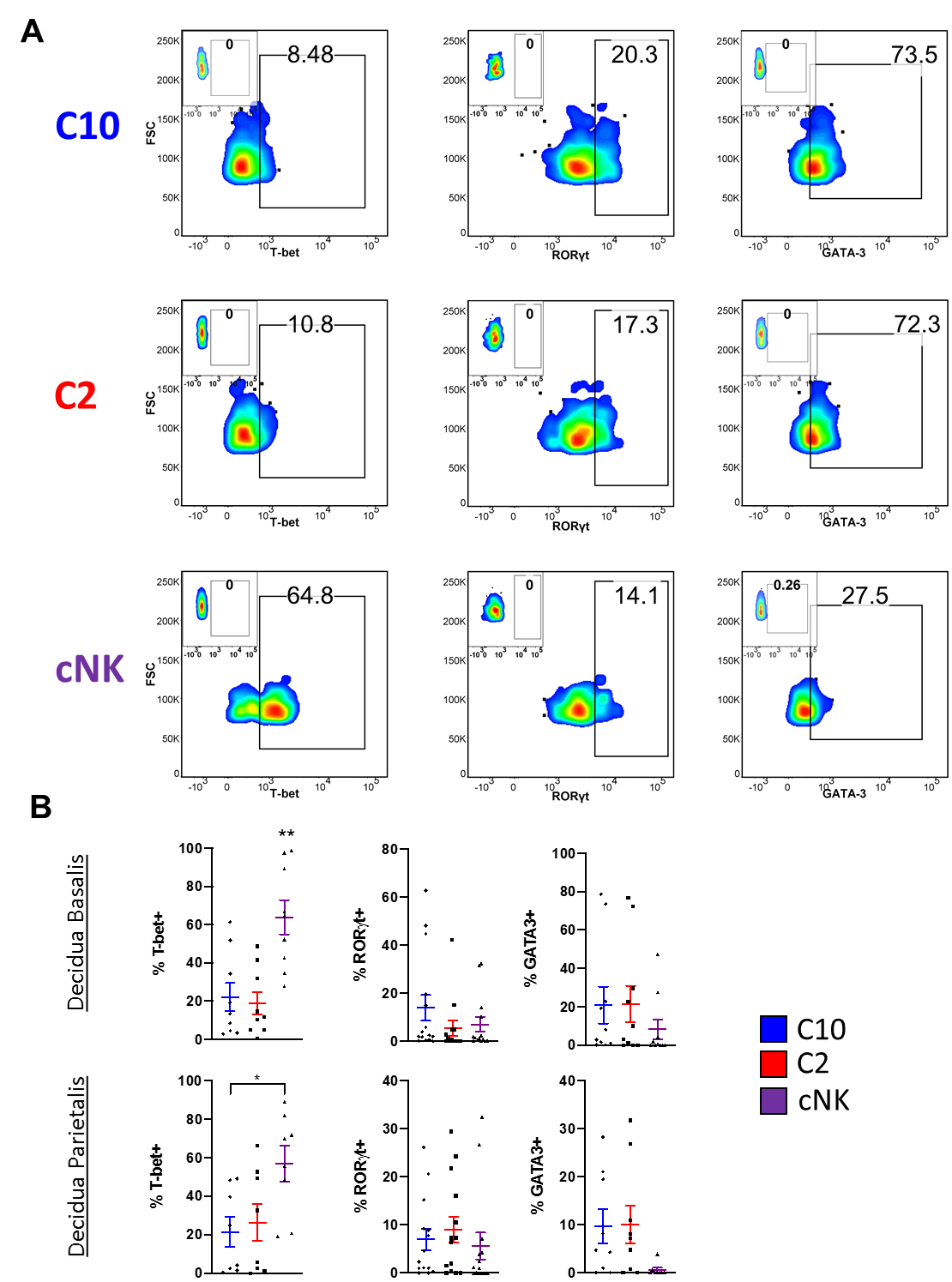


**Supplementary Figure 5.** Expression of transcription factor expression of T-bet, GATA-3, and RORγt. (A) Flow plots of pre-gated C10, C2, and cNK decidual ILCs and expression of T-bet, GATA-3, and RORγt. Data representative of 16 experiments. (B) Proportions of T-bet^+^, GATA-3^+^, and RORγt^+^ ILCs in the decidua basalis (top) and decidua parietalis (bottom). Decidua basalis, n = 10-15; decidua parietalis, n = 9-14. All data presented as mean ± SEM. Statistical significance was determined by ANOVA followed by Tukey test. *p < 0.05, **p < 0.005. FMOs are displayed for each subset and transcription factor.

**
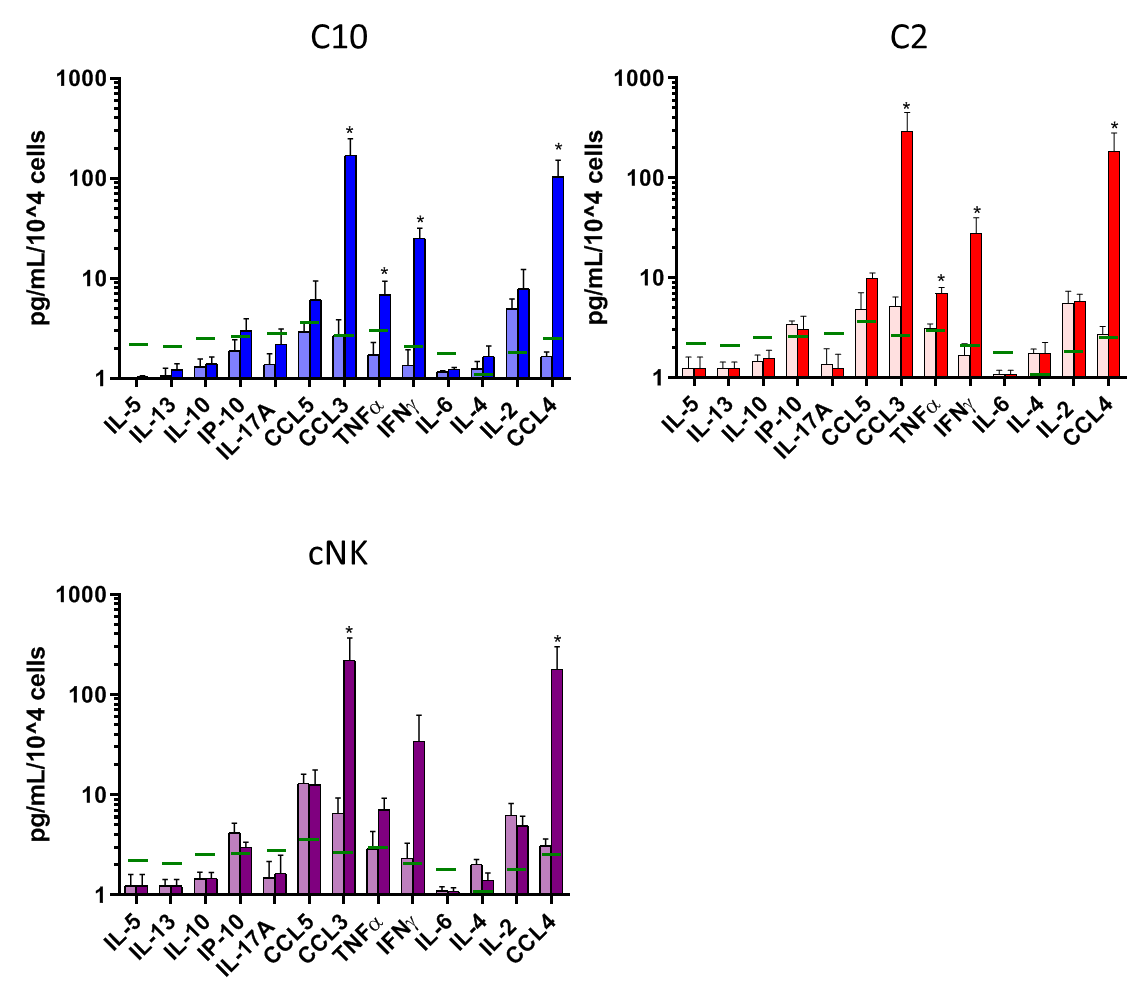
Supplementary Figure 6.** Cytokine Production Screen of PMA/Ionomycin-Stimulated C10, C2, and cNK. C10 and C2 were sorted from the decidua basalis, while cNKs were sorted from PBMCs. Cells were then activated with PMA/Ionomycin for 24 hours. Supernatants were then assessed using a cytometric bead analysis array. Light coloring indicates unstimulated control values, dark coloring indicated activated samples. n = 3. All data presented as mean ± SEM. Student’s t-test were used to compare unstimulated and PMA/Ionomycin-stimulated for each secreted factor (* p < 0.05). Green line represents the Minimum Detectable Concentration (MDC) as indicated by the manufacturer.


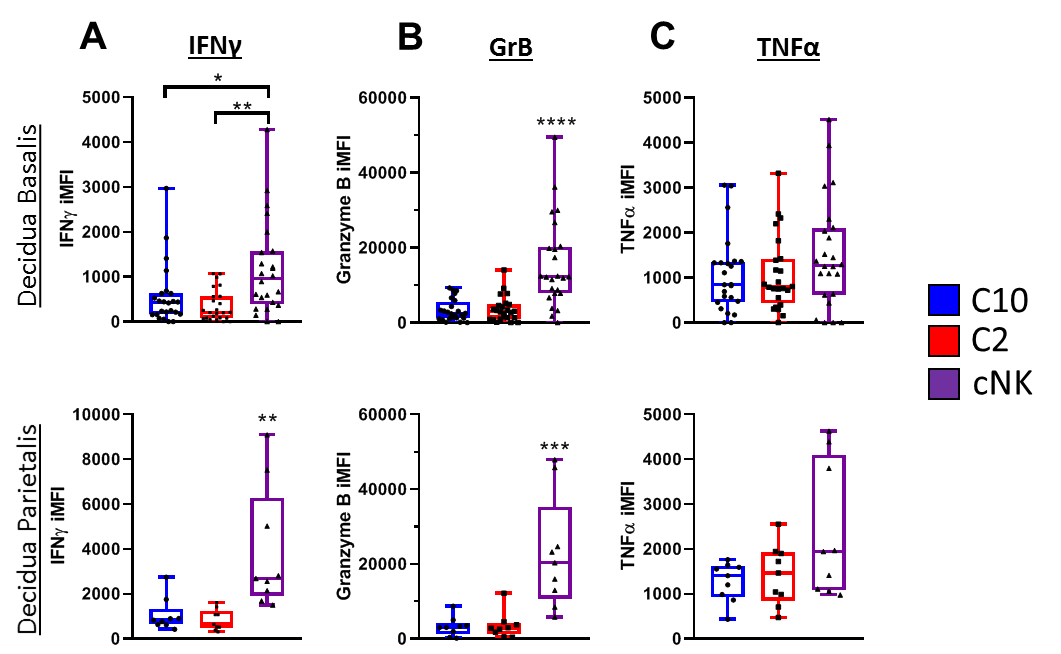


**Supplementary Figure 7.** Functional response of decidual ILCs. Integrated Mean Fluorescence Intensity (iMFI) was determined for C10, C2, and cNKs to assess total functional response. Decidua basalis, n = 24; decidua parietalis, n = 9. Data represented as max/min, median, and 25^th^ and 75^th^ percentiles. Statistical significance was determined by ANOVA, followed by Tukey post-hoc tests. *p < 0.05, **p < 0.005, ***p < 0.0005, ****p < 0.00001. *For statistical details see Supplementary Table 6.. Data is provided in Datasheet 1.*

A


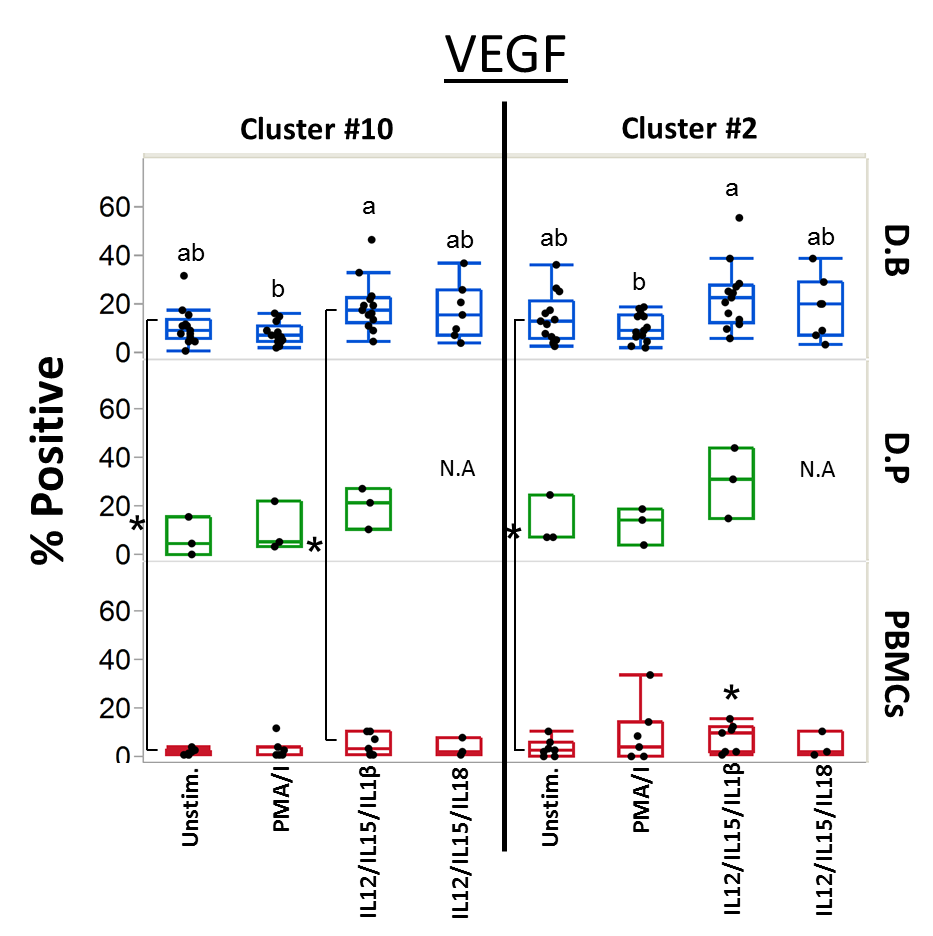


Unstimulated

PMA/I

B

**Supplementary Figure 8.** C10 and C2 LCs produce VEGF upon activation. Decidual ILCs were activated with PMA/Ionomycin and assessed for VEGF production. **(A)** Quantification of VEGF positive cells. Data represented as max/min, median, and 25^th^ and 75^th^ percentiles. Statistical significance was determined by ANOVA followed by Tukey test. Letters indicate comparison across cell types within tissues, with different letters indicating statistically differences (<0.05). * indicates differences within cell type across tissues. For B, unstim, PMA/Ionomycin, IL12/IL15/IL1β): Decidua basalis, n = 13; decidua parietalis, n = 3; PBMCs, n = 7. For (E, IL12/IL15/IL18): Decidua basalis, n = 7; PBMCs, n = 3. **(B)** Sorted C10 and C2 from the decidua basalis were activated with PMA/Ionomycin for 24 hours. Supernatants were then assessed using a cytometric bead analysis array. Light coloring indicates unstimulated control values, dark coloring indicated activated samples. n = 3. *For statistical details see Supplementary Table 9. Data is provided in Datasheet 1.*


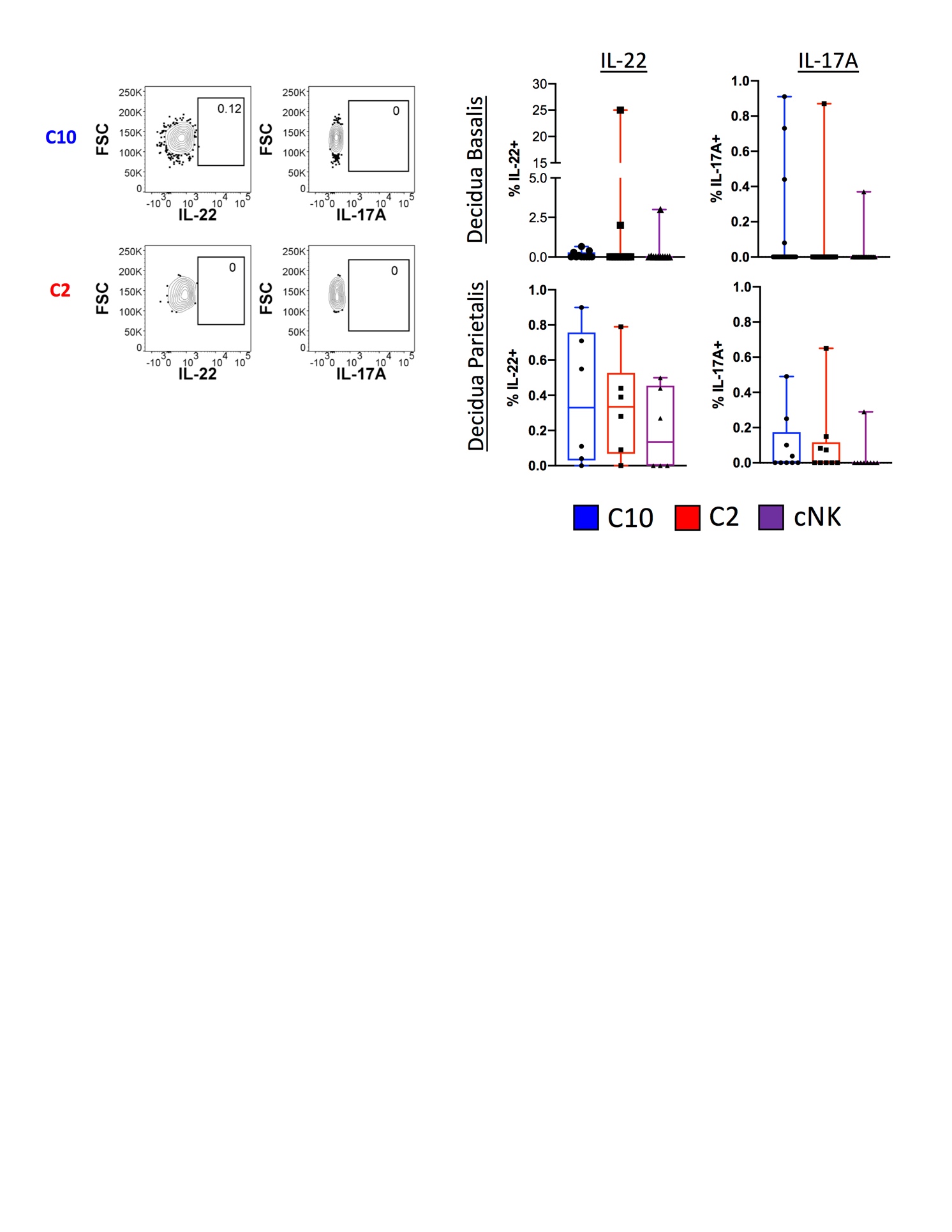


**Supplementary Figure 9.** Production of T_H_17-type cytokines by C10 and C2 dILCs. Decidual ILCs were activated with PMA/Ionomycin and the production of IL-22 and IL-17A was assessed. For IL-22: decidua basalis n = 11; decidua parietalis n = 6. For IL-17A, decidua basalis n = 24; decidua parietalis n = 9. *Data is provided in Datasheet 1.*

**
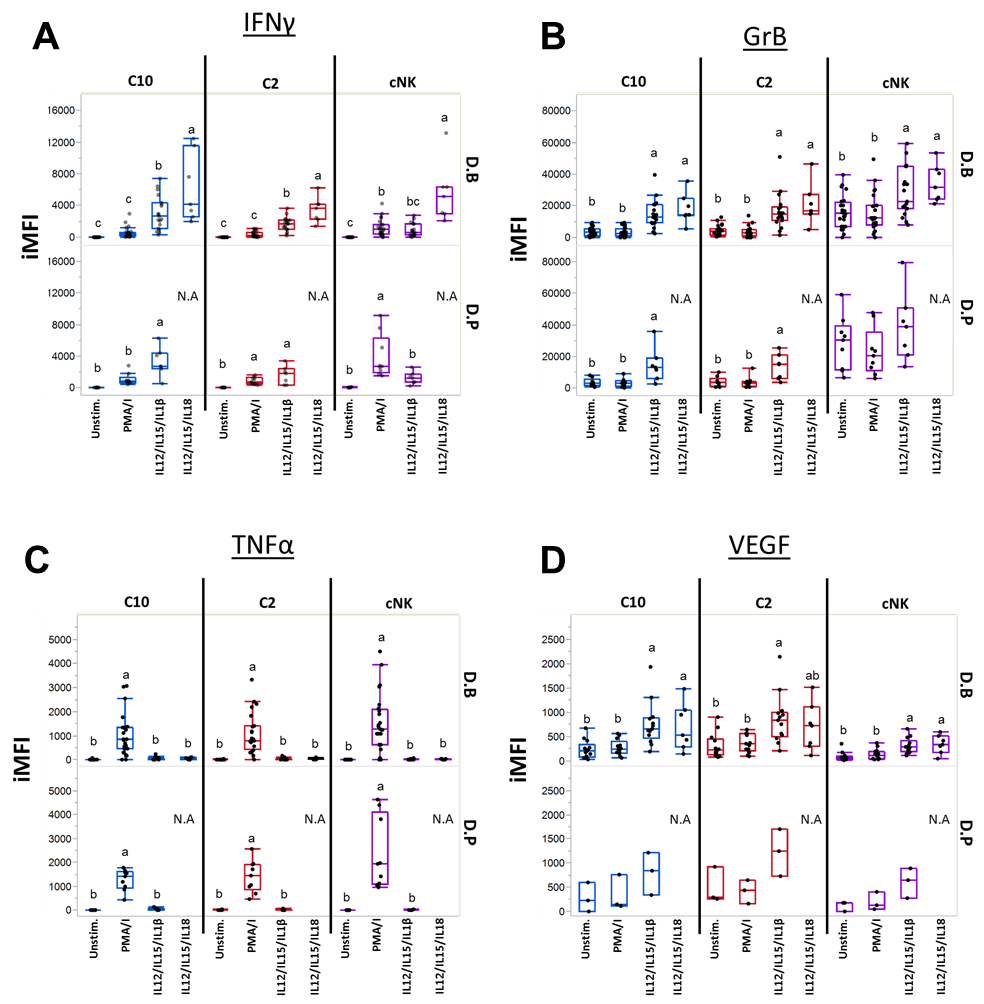
**

**Supplementary Figure 10** Functional response of unique decidual ILCs to cytokine stimulation. C10, C2, and cNKs were stimulated with PMA/Ionomycin, IL-12/IL-15/IL-1β, or IL-12/IL-15/IL-18 and the iMFI was calculated for **(A)** INFγ, **(B)** Granzyme B, **(C)** TNFα, and **(D)** VEGF to determine total functional response. Data represented as max/min, median, and 25^th^ and 75^th^ percentiles. Statistical significance was determined by ANOVA followed by Tukey test and are demonstrated by letters, with different letters indicating statistical differences within a subset (<0.05). Additionally, comparisons across subsets within treatments were performed, for simplicity p-values are found in supplementary Table 11. For (**A-C**, unstim and PMA/Ionomycin): Decidua basalis, n = 24; decidua parietalis, n = 9. For (**A-C**, IL12/IL15/IL1β): Decidua basalis, n = 19; decidua parietalis, n = 7 (**A-C**, IL12/IL15/IL18): Decidua basalis, n = 7. For (**D**, unstim, PMA/Ionomycin, IL12/IL15/IL1β): Decidua basalis, n = 13; decidua parietalis, n = 3. For (**D**, IL12/IL15/IL18): Decidua basalis, n = 7. N.A = not analyzed. *For statistical details see Supplementary Table 11. Data is provided in Datasheet 1.*


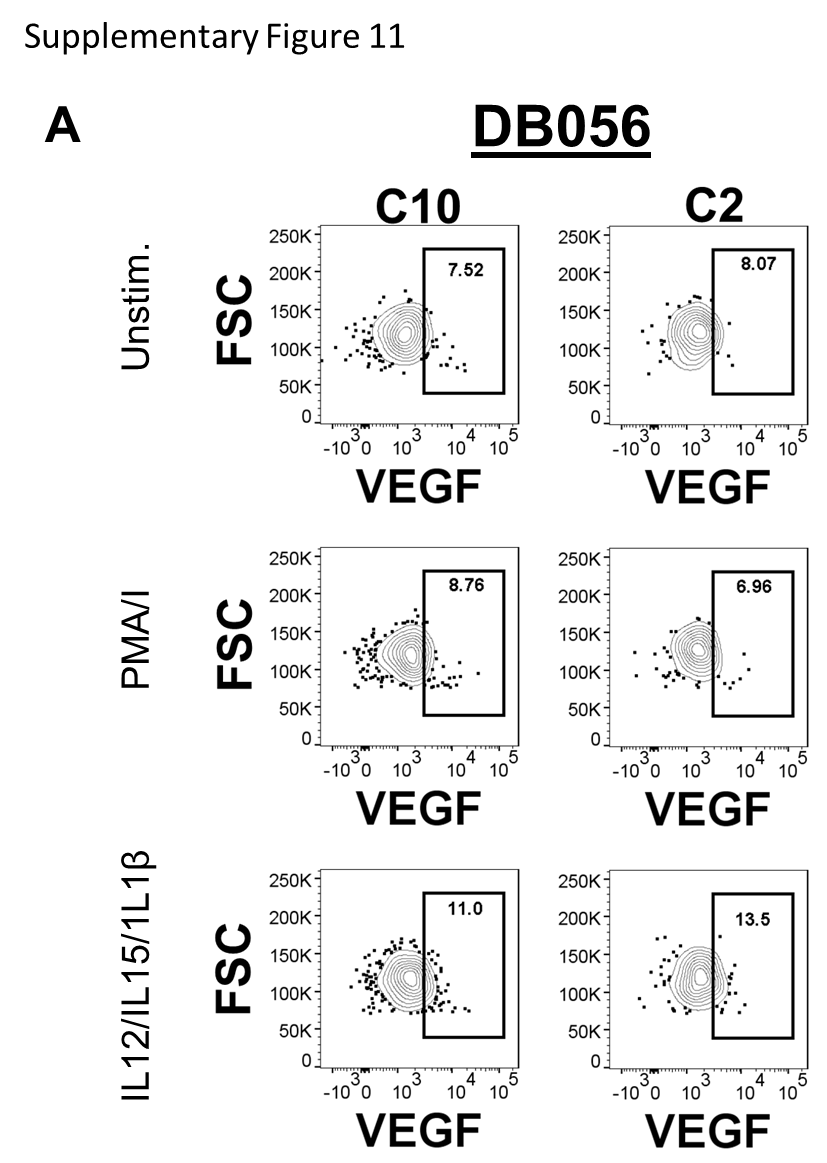


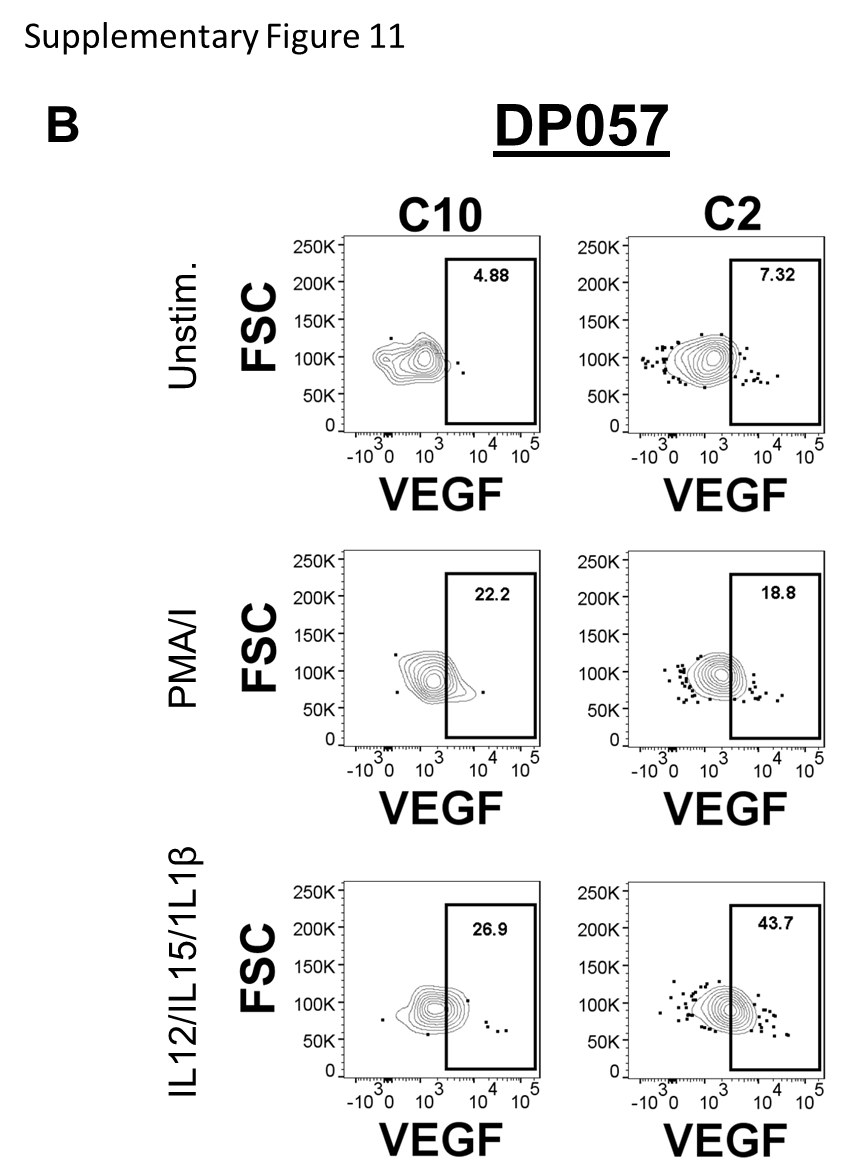


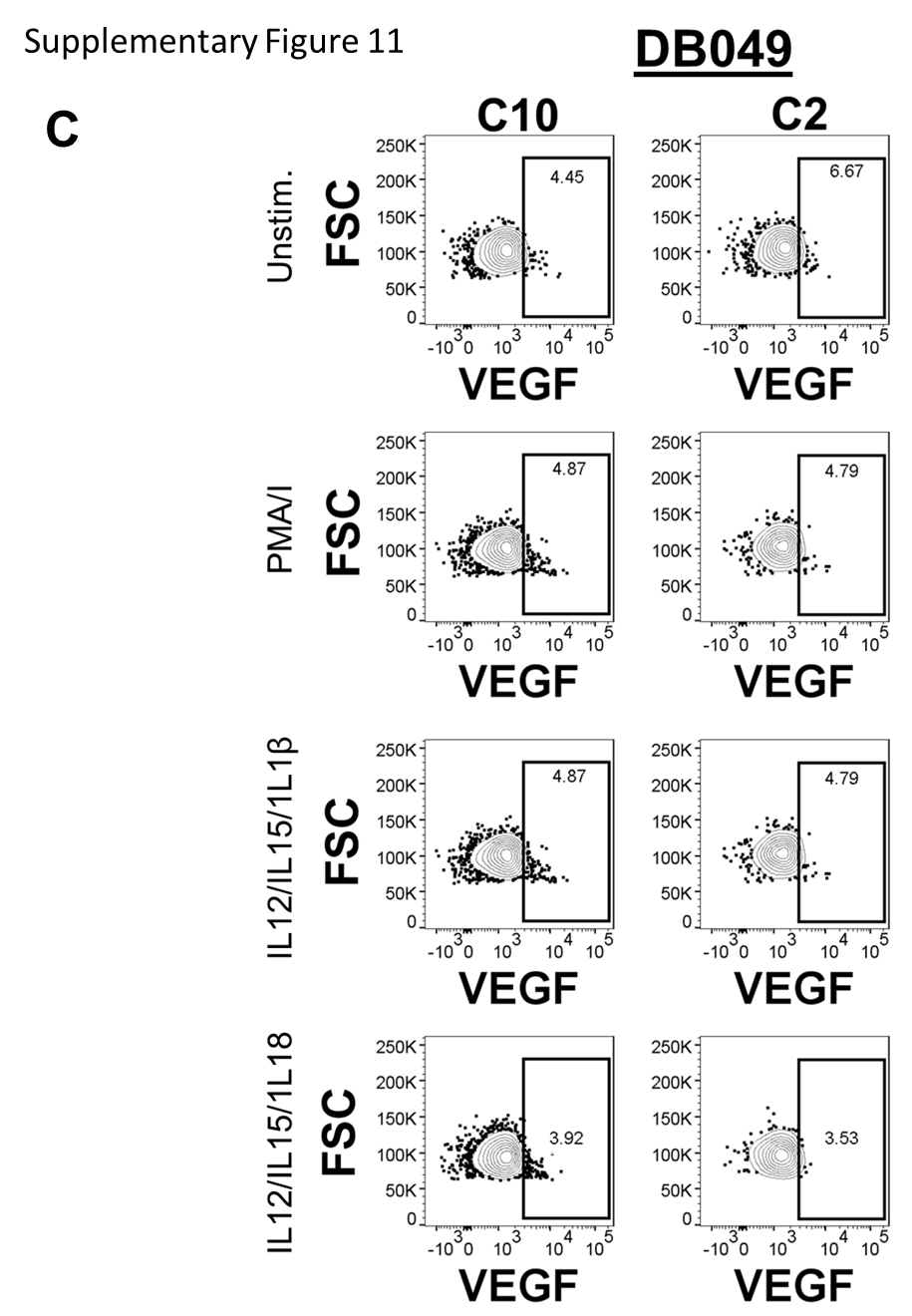

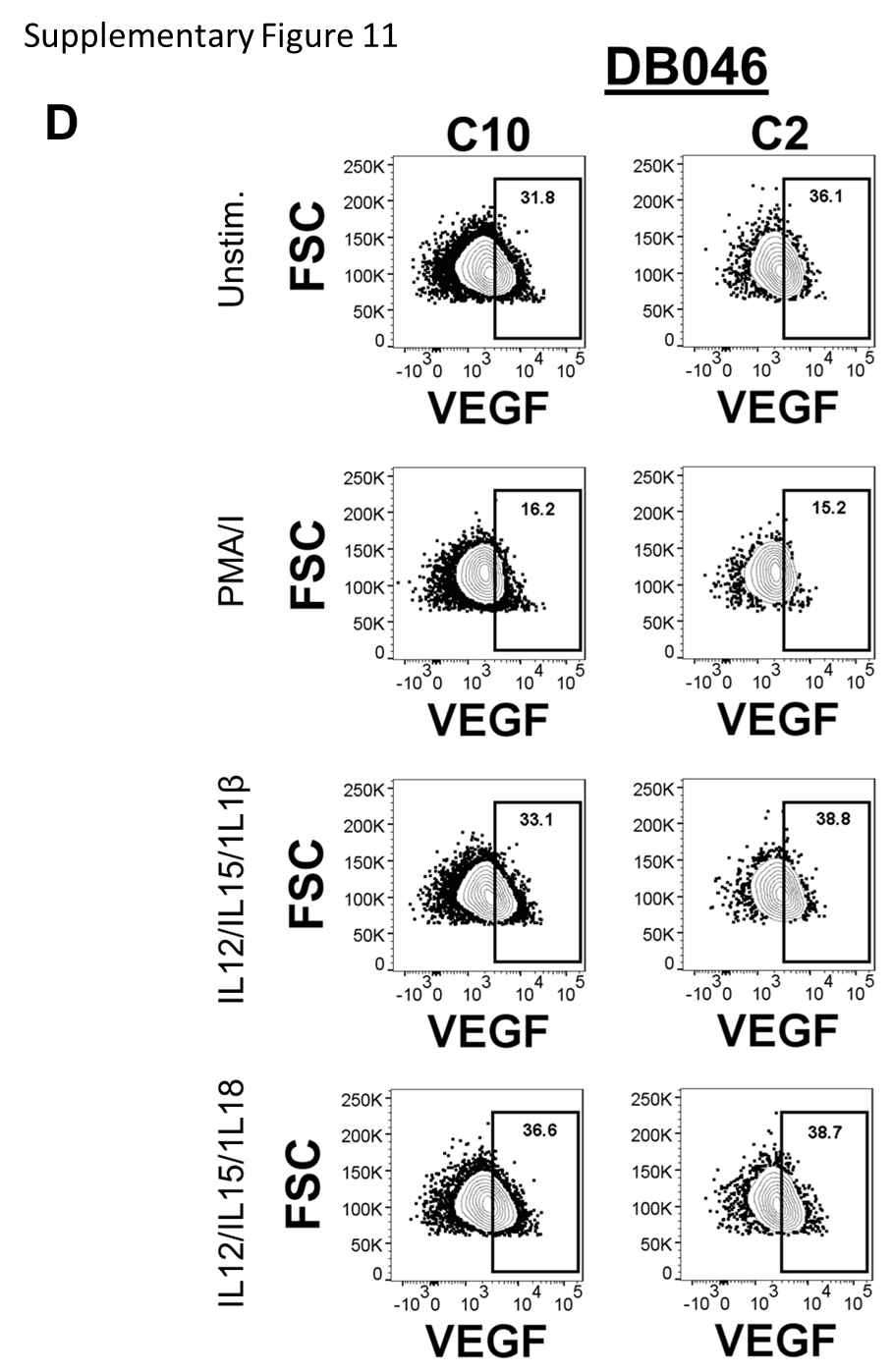


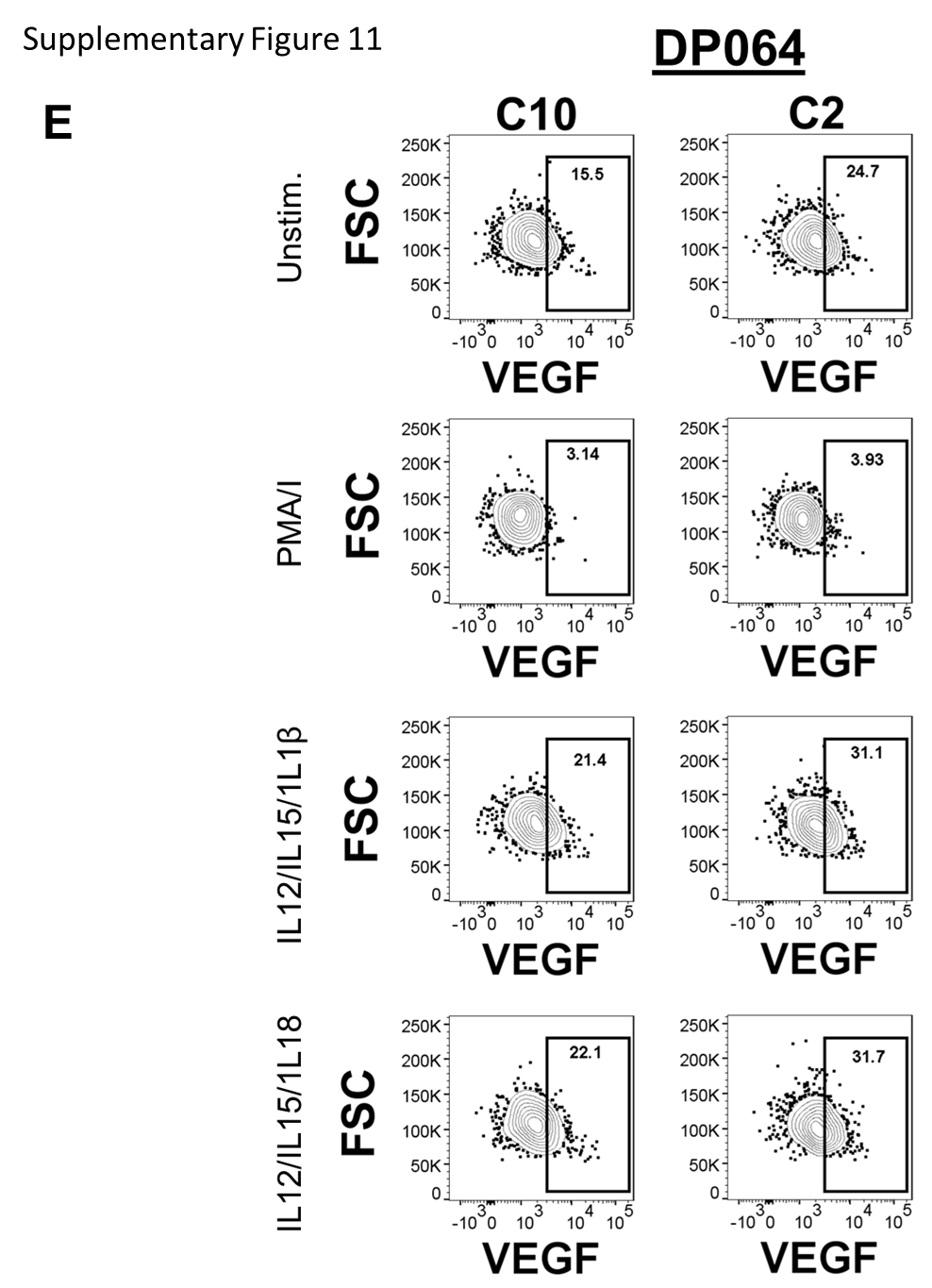


**Supplementary Figure 11. (A – E)** Representative plots assessing VEGF production across different treatments in 5 independent decidual samples from 2 independent experiments.


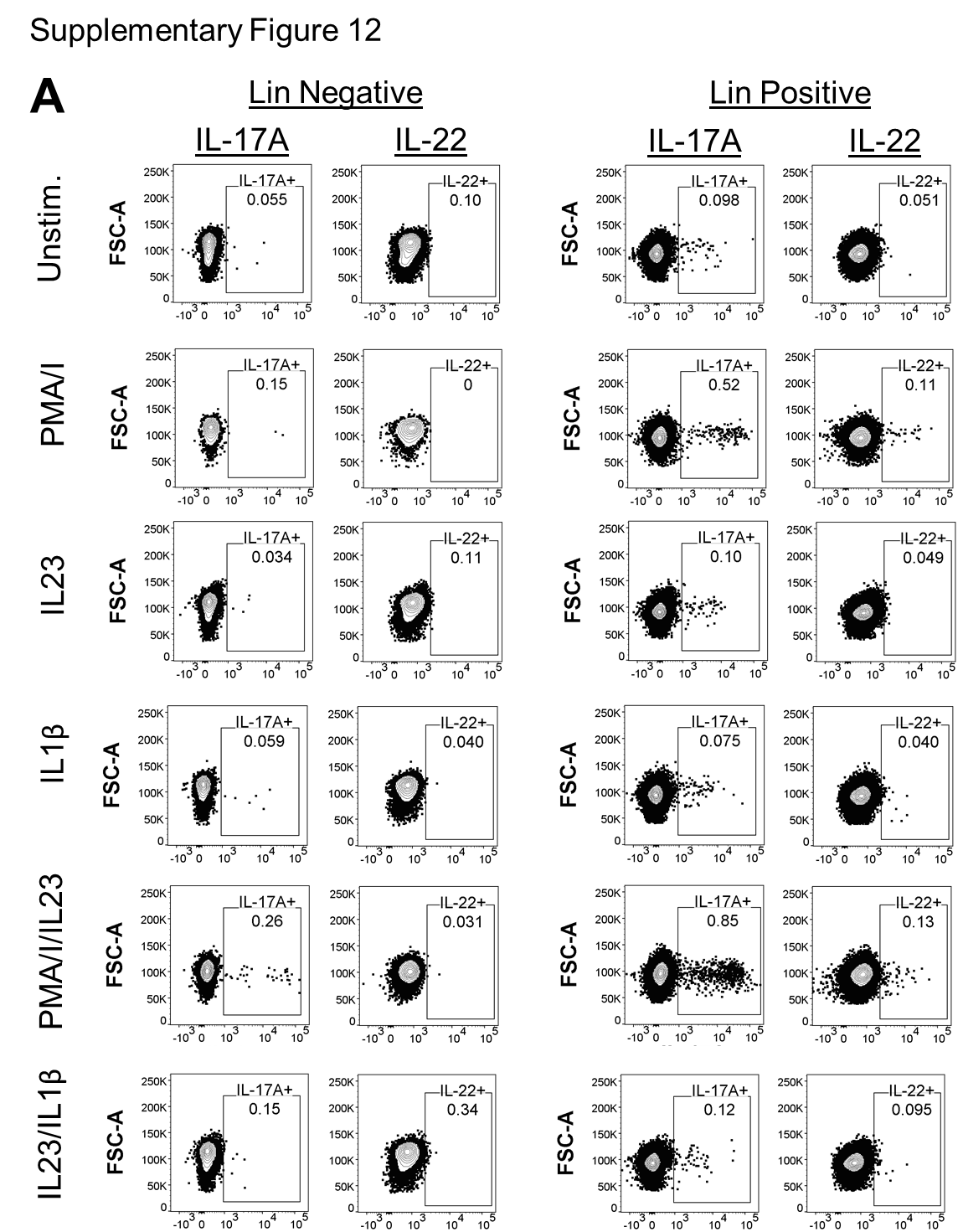


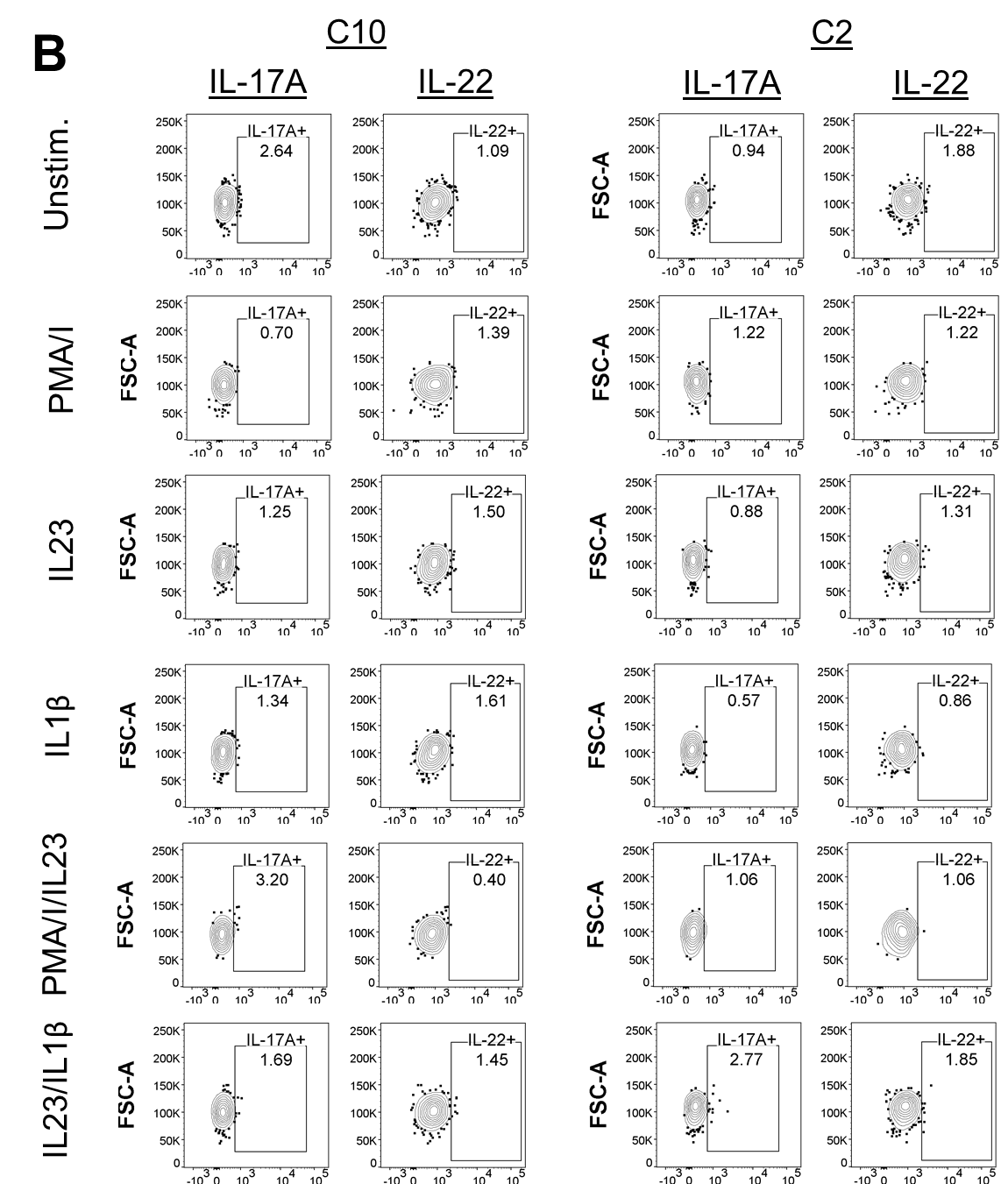


**Supplementary Figure 12.** Decidual ILCs C10 and C2 do not produce IL-17A nor IL-22. MCs were activated with PMA/Ionomycin, IL-23, IL-1β, PMA/Ionomycin/IL-23, or IL-23/IL-1β and expression of IL-17A and IL-22 were assessed via flow cytometry. Representative plots for **(A)** Lineage (CD3, CD14, CD19) Negative and Lineage Positive MCs from PBMCs and **(B)** Decidual basalis C10 and C2 ILCs. Representative of 3 decidual basalis samples.

Supplementary Table 1. Number of samples in each figure.

| **Supplementary Table 2.** Antibodies used for flow cytometry analysis. | | | | |  |
| --- | --- | --- | --- | --- | --- |
| **Marker** | **Clone** | **Fluorochrome** | **Catalog#** | **Supplier** | **Dilution** |
| CD117 | 104D2 | BB515 | 565172 | BD Bioscience | 1ul:100ul |
| CD117 | 104D2 | BV605 | 562687 | BD Bioscience | 1ul:100ul |
| CD127 | A019D5 | BV785 | 351330 | BioLegend | 1ul:100ul |
| CD14 | M5E2 | BV510 | 563079 | BD Bioscience | 0.5ul:100ul |
| CD16 | 3G8 | BUV496 | 564653 | BD Bioscience | 0.125ul:100ul |
| CD19 | SJ25C1 | APC-H7 | 560177 | BD Bioscience | 0.25ul:100ul |
| CD19 | SJ25C1 | BV510 | 562947 | BD Bioscience | 0.25ul:100ul |
| CD3 | UCHT-1 | BV510 | 563109 | BD Bioscience | 0.50ul:100ul |
| CD335 (NKp46) | 9E2/NKp46 | PE | 557991 | BD Bioscience | 0.25ul:100ul |
| CD34 | 581 | PE-Cy5 | 555823 | BD Bioscience | 0.25ul:100ul |
| CD45 | 2D1 | APC-H7 | 560178 | BD Bioscience | 0.25ul:100ul |
| CD45 | 2D1 | Alexa 700 | 368513 | BioLegend | 1ul:100ul |
| CD49a | TS2/7 | PE-Vio770 | 130-101-403 | Miltenyi | 1ul:100ul |
| CD56 | NCAM16.2 | BV421 | 562751 | BD Bioscience | 1ul:100ul |
| CD94 | HP-3D9 | PerCP-Cy5.5 | 562361 | BD Bioscience | 0.5ul:100ul |
| CXCR4 | 12G5 | BUV395 | 563924 | BD Bioscience | 0.5ul:100ul |
| Eomes | WD1928 | PE-eFluor610 | 61-4877-42 | eBioscience | 1ul:100ul |
| GATA-3 | 16E10A23 | PE | 653803 | BioLegend | 4ul: 100ul |
| Granzyme B | GB11 | Alexa 647 | 515405 | BioLegend | 2ul:100ul |
| IL-17A | BL168 | PE-Dazzle594 | 512335 | BioLegend | 1ul:100ul |
| IL-22 | 2612A41 | APC | 366705 | BioLegend | 2:100ul |
| IL-8 | E8N1 | Alexa 488 | 511411 | BioLegend | 1ul:100ul |
| IFNγ | 4S.B3 | Alexa 488 | 502517 | BioLegend | 2ul:100ul |
| RORγt | AFKJS-9 | APC | 17-6988-82 | eBioscience | 8ul:100ul |
| T-bet | O4-46 | BV650 | 564142 | BD Bioscience | 4ul:100ul |
| TNFα | MAb11 | BV650 | 502937 | BioLegend | 1:100ul |
| VEGF | 23410 | APC | IC2931A | R&D Systems | 1ul:100ul |

| **Supplementary Table 3.** Reagents used for activation assays | |  |  |
| --- | --- | --- | --- |
| **Cytokine** | **Catalog#** | **Supplier** | **Dilution or Final Concentration** |
| Recombinant Human IL-23 (carrier-free) | 574102 | BioLegend | 10 ng/mL |
| Recombinant Human IL-12(p70) (carrier-free) | 573002 | BioLegend | 50 ng/mL |
| Recombinant Human IL-15 (carrier-free) | 570306 | BioLegend | 50 ng/mL |
| Recombinant Human IL-18 (carrier-free) | 592102 | BioLegend | 50 ng/mL |
| Recombinant Human IL-1β (carrier-free) | 579402 | BioLegend | 50 ng/mL |
|  |  |  |  |
| Cell Activation Cocktail (without Brefaldin A) | 423301 | BioLegend | 1:500 |
| Leokocyte Activation Cocktail, with BD GolgiPlug™ | 550583 | BD Bioscience | 1:500 |
| BD GolgiPlug™ (Brefaldin A) | 555029 | BD Bioscience | 1:1000 |
| BD GolgiStop™ (Monensin) | 554724 | BD Bioscience | 1:1000 |

**Supplementary Table 4.** Presumed classification of ILC subsets.

| **Cluster** | **Phenotype** | **Presumed Name** |
| --- | --- | --- |
| 1 | CD16^-^Tbet^-^CD127^+^RORγt^-^CD117^+^CD49a^-^CD94^-^CD335^-^Eomes^-^ | ILC3 |
| 2 | CD16^-^Tbet^-^RORγt^-^CXCR4^+^CD49a^+^CD56^+^CD94^+^Eomes^-^ | CD56 Bright NK |
| 3 | CD16^-^Tbet^-^CD127^-^RORγt^+^CXCR4^+^CD49a^-^CD56^-^CD94^-^CD335^-^Eomes^-^ | LTi-like |
| 4 | CD16^-^Tbet^-^CD127^+^CD117^-^CD49a^+^CD56^+^CD94^+^CD335^+^Eomes^+^ | CD56 Bright NK |
| 5 | CD16^-^Tbet^-^CD127^-^CD117^-^CD49a^+^CD56^+^CD94^+^CD335^+^Eomes^+^ | CD56 Bright NK |
| 6 | CD16^-^Tbet^-^CD127^+^RORγt^+^CXCR4^-^CD56^-^CD94^-^CD335^-^Eomes^-^ | LTi-like |
| 7 | CD16^+^Tbet^+^CD127^+^CD117^-^CD56^-^CD94^-^Eomes^+^ | CD56 Dim NK |
| 8 | Tbet^+^RORγt^-^CXCR4^-^CD56^-^CD94^-^CD335^-^ | ILC1 |
| 9 | CD16^+^Tbet^+^CD127^-^CD117^-^CD49a^+^CD56^-^CD94^-^CD335^-^Eomes^+^ | CD56 Dim NK |
| 10 | CD16^-^Tbet^-^CD127^-^RORγt^+^CD117^-^CXCR4^+^CD49a^-^CD56^+^CD94^+^CD335^+^Eomes^+^ | CD56 Bright NK |
| 11 | Tbet^+^CD127^-^RORγt^-^CD117^-^CXCR4^-^CD49a^-^CD56^-^CD94^-^CD335^-^Eomes^-^ | ILC1 |
| 12 | CD16^+^Tbet^+^CD127^+^RORγt^-^CD117^-^CXCR4^-^CD49a^-^CD56^-^CD94^-^Eomes^+^ | CD56 Dim NK |
| 13 | CD16^+^Tbet^+^CD127^-^RORγt^-^CD117^-^CXCR4^-^CD49a^-^CD56^-^CD94^-^Eomes^+^ | CD56 Dim NK |

| **Supplementary Table 5.** Statistical results related to Figure 5. | | | | |  |  |
| --- | --- | --- | --- | --- | --- | --- |
|  |  | **ANOVA** | | | **Tukey's post-hoc** | |
| **Tissue** | **Cytokine** | **F value** | **D.F** | **p-value** | **Comparison** | **p-value** |
| D.B | IFN𝛾 | 8.379 | 2,69 | **0.0006** | C10 vs C2 | 0.6533 |
|  |  |  |  |  | C10 vs cNK | **0.0098** |
|  |  |  |  |  | C2 vs cNK | **0.0006** |
|  | IL-8 | 0.5759 | 2,69 | 0.5649 |  |  |
|  |  |  |  |  |  |  |
|  |  |  |  |  |  |  |
|  | GrB | 22.02 | 2,69 | **<0.0001** | C10 vs C2 | 0.7091 |
|  |  |  |  |  | C10 vs cNK | **<0.0001** |
|  |  |  |  |  | C2 vs cNK | **<0.0001** |
|  | TNF⍺ | 0.7459 | 2,69 | 0.4781 |  |  |
|  |  |  |  |  |  |  |
|  |  |  |  |  |  |  |
| D.P | IFN𝛾 | 8.455 | 2,24 | **0.0017** | C10 vs C2 | 0.9564 |
|  |  |  |  |  | C10 vs cNK | **0.0063** |
|  |  |  |  |  | C2 vs cNK | **0.0031** |
|  | IL-8 | 0.1481 | 2,24 | 0.8632 |  |  |
|  |  |  |  |  |  |  |
|  |  |  |  |  |  |  |
|  | GrB | 7.876 | 2,24 | **0.0023** | C10 vs C2 | 0.9562 |
|  |  |  |  |  | C10 vs cNK | **0.0043** |
|  |  |  |  |  | C2 vs cNK | **0.0084** |
|  | TNF⍺ | 3.798 | 2,24 | **0.0369** | C10 vs C2 | 0.9409 |
|  |  |  |  |  | C10 vs cNK | **0.0461** |
|  |  |  |  |  | C2 vs cNK | 0.0910 |

D.B = decidua basalis; D.P = decidua parietalis

| **Supplementary Table 6.** Statistical results related to Supplementary Figure 7. | | | | | |  |
| --- | --- | --- | --- | --- | --- | --- |
|  |  | **ANOVA** | | | **Tukey's post-hoc** | |
| **Tissue** | **Cytokine** | **F value** | **D.F** | **p-value** | **Comparison** | **p-value** |
| D.B | INF𝛾 | 7.505 | 2,69 | **0.0011** | C10 vs C2 | 0.5948 |
|  |  |  |  |  | C10 vs cNK | **0.0200** |
|  |  |  |  |  | C2 vs cNK | **0.0011** |
|  | GrB | 21.58 | 2,69 | **<0.0001** | C10 vs C2 | 0.9925 |
|  |  |  |  |  | C10 vs cNK | **<0.0001** |
|  |  |  |  |  | C2 vs cNK | **<0.0001** |
|  | TNF⍺ | 1.623 | 2,69 | 0.2047 |  |  |
|  |  |  |  |  |  |  |
|  |  |  |  |  |  |  |
| D.P | INF𝛾 | 9.538 | 2,24 | **0.0009** | C10 vs C2 | 0.9554 |
|  |  |  |  |  | C10 vs cNK | **0.0037** |
|  |  |  |  |  | C2 vs cNK | **0.0018** |
|  | GrB | 13.88 | 2,24 | **<0.0001** | C10 vs C2 | 0.998 |
|  |  |  |  |  | C10 vs cNK | **0.0003** |
|  |  |  |  |  | C2 vs cNK | **0.0004** |
|  | TNF⍺ | 3.31 | 2,24 | 3.310 |  |  |
|  |  |  |  |  |  |  |
|  |  |  |  |  |  |  |

| **Supplementary Table 7.** Statistical results related to Figure 6B. | | | | |  |  |
| --- | --- | --- | --- | --- | --- | --- |
|  |  | **ANOVA** | | | **Tukey's post-hoc** | |
| **Tissue** | **Cytokine** | **F value** | **D.F** | **p-value** | **Comparison** | **p-value** |
| D.B | VEGF | 6.57 | 2,36 | **0.0037** | C10 vs C2 | 0.3367 |
|  |  |  |  |  | C10 vs cNK | 0.0902 |
|  |  |  |  |  | C2 vs cNK | **0.0027** |
| D.P | VEGF | 0.5052 | 2,6 | 0.6270 |  |  |
|  |  |  |  |  |  |  |
|  |  |  |  |  |  |  |

D.B = decidua basalis; D.P = decidua parietalis

| **Supplementary Table 8.** Statistical results related to Figure 6C. | | | |  |
| --- | --- | --- | --- | --- |
| **ANOVA** | | | **Tukey's post-hoc** | |
| **F value** | **D.F** | **p-value** | **Comparison** | **p-value** |
| 33.67 | 2,12 | **<0.0001** | Unstim C10 v. Stim C10 | **0.0086** |
|  |  |  | Unstim C2 v. Stim C2 | 0.9629 |
|  |  |  | Unstim cNK v. Stim cNK | >0.9999 |
|  |  |  | Unstim C10 v. Unstim C2 | **0.0002** |
|  |  |  | Unstim C10 v. Unstim cNK | 0.9451 |
|  |  |  | Unstim C2 v. Unstim cNK | **<0.0001** |
|  |  |  | Stim C10 v. Stim C2 | 0.9995 |
|  |  |  | Stim C10 v. Stim cNK | **0.0103** |
|  |  |  | Stim C2 v. Stim cNK | **0.0061** |

Supplementary Table 9. Statistical results related to Supplementary Figure 8A.

Supplementary Table 10. Statistical results related to Figure 7.

**Supplementary Table 11.** Statistical results related to Supplementary Figure 10.

Supplementary Table 12. Statistical results related to Figure 8.

Supplementary Table 13. Statistical results related to Figure 9.
